# Macrophage-derived oncostatin M repairs the lung epithelial barrier during inflammatory damage

**DOI:** 10.1126/science.adi8828

**Published:** 2025-07-10

**Authors:** Daisy A. Hoagland, Patricia Rodríguez-Morales, Alexander O. Mann, Alan Y. Baez Vazquez, Shuang Yu, Alicia Lai, Harry Kane, Susanna M. Dang, Yunkang Lin, Louison Thorens, Shahinoor Begum, Martha A. Castro, Scott D. Pope, Jaechul Lim, Shun Li, Xian Zhang, Ming O. Li, Carla F. Kim, Ruaidhrí Jackson, Ruslan Medzhitov, Ruth A. Franklin

**Affiliations:** 1 Department of Stem Cell and Regenerative Biology, Harvard University; Cambridge, MA, USA.; 2 Department of Immunology, Harvard Medical School; Boston, MA, USA.; 3 Department of Immunobiology, Yale University School of Medicine; New Haven, CT, USA.; 4 Stem Cell and Regenerative Biology Program, Department of Pediatrics, Divisions of Hematology/Oncology and Pulmonary Medicine, Boston Children’s Hospital, Boston, MA, USA.; 5 Department of Genetics, Harvard Medical School, Boston, MA, USA.; 6 Institute for Mechanobiology, Department of Bioengineering, College of Engineering, Northeastern University; Boston, MA, USA; 7 Immunology Program, Sloan Kettering Institute, Memorial Sloan Kettering Cancer Center; New York, NY, USA.; 8 Harvard Stem Cell Institute; Cambridge, MA, USA; 9 Howard Hughes Medical Institute; New Haven, CT, USA.; 10 Tananbaum Center for Theoretical and Analytical Human Biology, Yale University School of Medicine; New Haven, CT, USA.

## Abstract

Tissue repair programs must function alongside antiviral immunity to restore the lung epithelial barrier following infection. We found that macrophage-derived oncostatin M (OSM) counteracted the pathological effects of type I interferon (IFN-I) during infection and damage in mice. At baseline, OSM-deficient mice exhibited altered alveolar type II (ATII) epithelial cell states. In response to influenza or viral mimic challenge, mice lacking OSM exhibited heightened IFN-I responses and increased mortality. OSM delivery to the lung induced ATII proliferation and was sufficient to protect deficient mice against morbidity. Furthermore, OSM promoted organoid formation despite the growth-inhibitory effects of IFN-I. These findings identify OSM as an indispensable macrophage-derived growth factor that maintains the homeostasis of lung epithelial cells and promotes their proliferation to overcome IFN-I-mediated immunopathology.

## One sentence summary

Oncostatin M production by macrophages is required to regulate lung epithelial cell states and restore the epithelial barrier following viral-induced immunopathology.

Tissue damage is an inevitable consequence of infection, requiring repair programs to function alongside strategies to restrict pathogen spread. Mucosal tissues such as the respiratory tract are exposed to the external environment, necessitating robust inflammatory responses to fight infections. However, inflammation can also cause tissue damage, compromising epithelial barrier integrity and function. For example, type I interferons (IFN-I), which are essential antiviral mediators, can exacerbate lung pathology by driving immune cell infiltration, inducing epithelial cell death, and suppressing epithelial proliferation (1-4). Despite virus-mediated cell death and the inhibitory effects of IFN-I on proliferation, the epithelial barrier is rapidly restored after viral respiratory infections, suggesting the existence of mechanisms that counteract the impacts of IFN-I to maintain lung function.

We predicted that macrophages—innate immune cells involved in pathogen defense, tissue repair, and homeostasis (5-9)—may provide crucial signals that limit the detrimental effects of antiviral responses and facilitate epithelial repair in the damaged lung. Alveolar macrophages (AMs) reside within lung alveoli, where they interact with alveolar type I (ATI) and type II (ATII) epithelial cells (10). ATIs facilitate gas exchange, and ATIIs produce surfactants and serve as progenitors for both epithelial cell types (11). Given their strategic positioning and immunomodulatory functions, we hypothesized that a macrophage-derived factor may contribute to epithelial homeostasis and repair after viral infection. We focused on the cytokine oncostatin M (OSM), which is implicated in the pathogenesis of chronic inflammatory diseases such as inflammatory bowel disease, pulmonary fibrosis, rheumatoid arthritis, and cancer (12-14). However, its functions at steady state and in response to acute inflammatory challenges remain poorly understood.

## Results

### OSM is required for maintenance of ATII transcriptional states and for host survival during IAV infection

**Fig 1.**
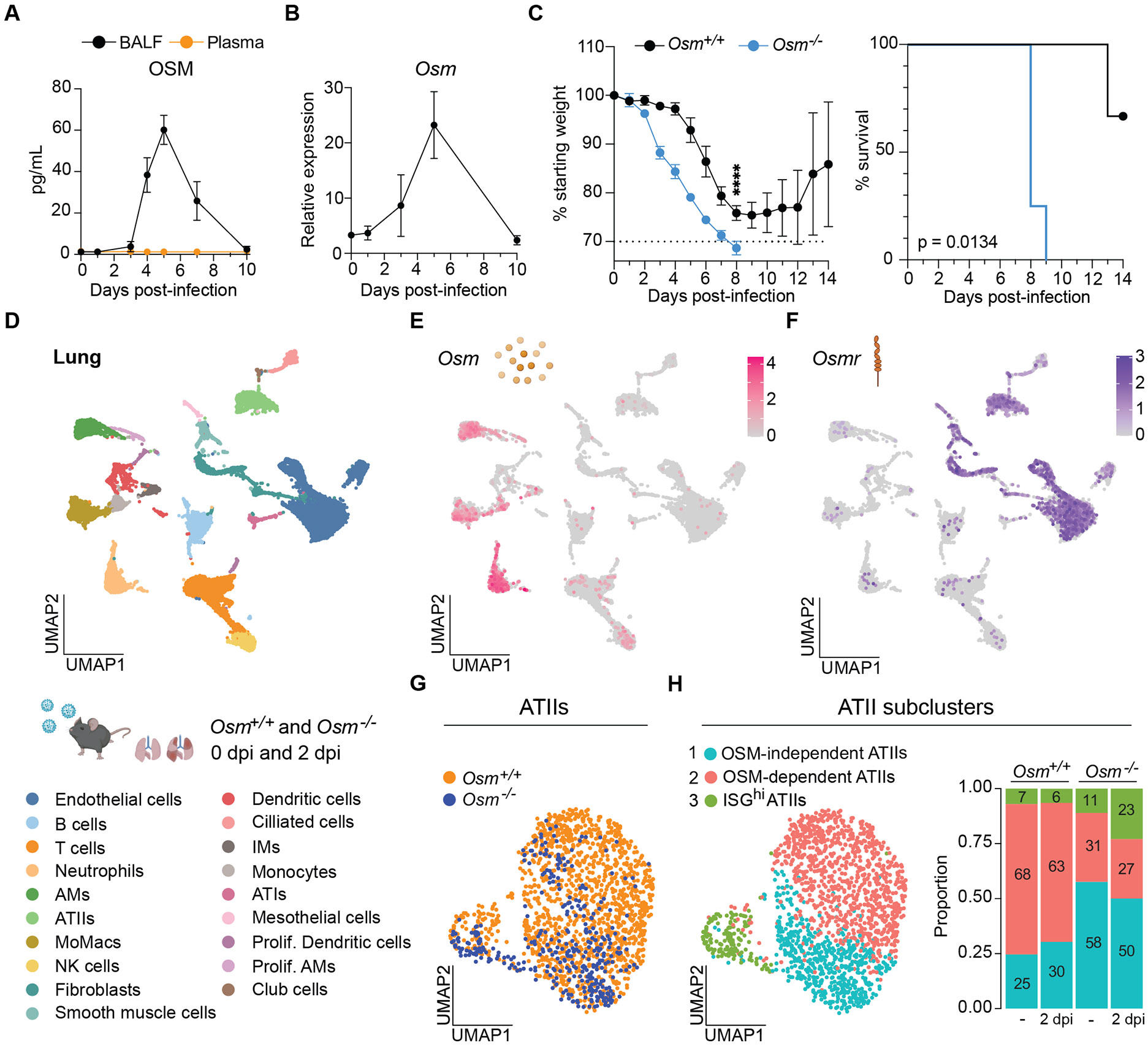
OSM is required for maintenance of ATII epithelial cell transcriptional states and for host survival during IAV infection. (**A** to **C**) Mice were infected intranasally (i.n.) with 225 to 300 plaque-forming units (PFU) of A/WSN/1933 (H1N1). BALF and plasma were collected at indicated time points, and OSM protein levels were assessed by enzyme-linked immunosorbent (ELISA) (A) (n = 3 mice per group). Whole-lung RNA was collected at indicated time points and *Osm* transcript expression measured by reverse transcription quantitative polymerase chain reaction (RT-qPCR) (B) (n = 3 mice per group). Percent initial body weight (left panel) and survival curve (right panel) after infection (C) (n = 3 to 4 mice per group); representative of at least two independent experiments. (**D** to **H**) Mice were infected i.n. with 225 PFU of A/WSN/1933 (H1N1) or mock-infected with PBS. Lungs were collected from IAV-infected mice at 2 days post-infection (2 dpi) and from mock-infected mice (0 dpi) for scRNA-seq analysis (n = 2 mice per group). Uniform manifold approximation and projection (UMAP) clustering and cell cluster annotation of scRNA-seq data from the lungs of mock-infected (0 dpi) and IAV-infected mice at 2 dpi (26,978 total cells) (D). *Osm* expression in annotated cell clusters in wild-type mice (15,349 cells) (E). *Osmr* expression in annotated cell clusters in wild-type mice (F). UMAP of re-clustered ATII subpopulations across genotypes (G). UMAP of reclustered ATII subpopulations in all mice (left panel) and stacked bar plot of ATII subpopulation percentages at baseline or 2 dpi across genotypes (right panel) (H). Female mice were used for experiments in this figure. Symbols represent mean data, with error bars indicating SD; ****P ≤ 0.0001, Two-way analysis of variance (ANOVA) up to day 8 for (C), left panel; log-rank Mantel-Cox test for (C), right panel. Scale bar represents log-normalized gene expression for (E) and (F).

At baseline, OSM was present in the lungs of wild-type (*Osm*^+/+^) mice but was undetectable in the lungs of OSM-deficient (*Osm*^−/−^) mice (fig. S1A). To investigate the role of OSM in acute inflammation and tissue damage, we utilized a sublethal model of influenza A virus (IAV) infection (A/WSN/1933; H1N1) (fig. S1B). We measured OSM levels in bronchoalveolar lavage fluid (BALF) and plasma of infected wild-type mice and detected only local production (Fig. 1A). OSM levels in BALF and transcript expression in the lung both peaked at 5 days post-infection (dpi) (Fig. 1, A and B).

Because OSM levels increased during IAV infection, we hypothesized that OSM may contribute to antiviral host defense. We infected *Osm*^+/+^ and *Osm*^−/−^ mice and found that *Osm*^−/−^ mice succumbed to sublethal IAV infection (Fig. 1C). Given that OSM was detectable at baseline and that weight curves diverged early during infection, we investigated the role of OSM in the lung at steady state and 2 dpi by performing single-cell RNA sequencing (scRNA-seq) on cells isolated from the lungs of *Osm*^+/+^ and *Osm*^−/−^ mice (Fig. 1D, fig. S1C, and data S1). *Osm* expression was detected primarily in myeloid subsets including AMs, monocytes, and neutrophils in wild-type mice (Fig. 1E). We reanalyzed publicly available datasets to examine the expression of the gene encoding OSM (*OSM)* and its coreceptor OSMR (*OSMR)* (fig. S2). *OSM* was found to be robustly expressed in myeloid subsets from dissociated lung samples of COVID-19 patients (fig. S2, A and B). These data suggested that *Osm* expression in myeloid cells was conserved across mice and humans during viral respiratory infections.

To evaluate macrophage production of OSM in response to diverse stimuli, we treated bone marrow-derived macrophages (BMDMs) with various stressors and pathogen-associated molecular patterns (PAMPs) *in vitro* and found heightened OSM levels after treatment (fig. S3A and B). We next stimulated AMs with the PAMP poly(I:C) *ex vivo*. Poly(I:C) mimics a double-stranded RNA viral replication product and stimulates TLR3 and RIG-I/MDA5 pathways (15). Unlike what we observed in BMDMs, poly(I:C) alone was insufficient to induce OSM production in AMs (fig. S3C). GM-CSF, a growth factor and ATII-derived signal essential for AM maintenance (16, 17), was required for OSM production, and its combination with poly(I:C) enhanced OSM levels (fig. S3C). To determine whether ontogeny influences OSM production, we stimulated fetal-derived AMs and monocyte-derived AMs (moAMs) *ex vivo*. AM origin was determined by using Siglec-F expression (18). We found that moAMs produced higher levels of OSM compared with fetal-derived AMs (fig. S3, D and E). Therefore, although both populations produce OSM, moAMs may serve as a major source of OSM during challenge. Altogether, these data demonstrated that OSM was produced by myeloid cells at steady state and elicited in response to infection and environmental stressors.

Expression of *Osmr* was restricted to non-immune populations including epithelial cells, fibroblasts, and endothelial cells in wild-type mice (Fig. 1F). Analysis of *OSMR* expression in healthy and COVID-19 patients (19, 20) showed similar expression patterns (fig. S2, A to C). *Ex vivo* stimulation of mouse lung cells with OSM, followed by analysis of downstream STAT3 phosphorylation, revealed robust responses in epithelial and stromal cells, with no substantial response detected in endothelial or immune cells (fig. S4). Given that the lung epithelium exhibited the greatest responsiveness to OSM, we assessed airway epithelial cells and ATIIs but found no defects in distribution or numbers in *Osm*^−/−^ mice (fig. S5). Considering the susceptibility of ATIIs to IAV infection (21), their critical role in maintaining and replenishing the alveolar epithelium (11), and their close proximity to OSM-producing AMs, we next examined ATII transcriptional profiles in the absence of OSM. Gene expression analysis validated our ATII annotation, showing high *Sftpc* expression compared with airway epithelial markers (*Scgb1a1* and *Foxj1*), together with ATII signature enrichment (fig. S6, A and B). Subclustering of ATIIs (fig. S6C) revealed three populations: a cluster predominant in *Osm*^−/−^ mice (cluster 1; OSM-independent), a cluster enriched in *Osm*^+/+^ mice (cluster 2; OSM-dependent), and a cluster characterized by expression of interferon stimulated genes (ISGs) (cluster 3; ISG^hi^) (Fig. 1, G and H, and data S2). Although cluster 1 showed no specific pathway enrichment, cluster 2 was enriched in protein translation pathways and cluster 3 in interferon signaling (fig. S6D and data S2). These data indicated that OSM shaped the expression profile of epithelial cells at baseline and during early IAV infection. Notably, at 2 dpi, cluster 3 (ISG^hi^) was enriched in *Osm*^−/−^ mice, suggesting an exacerbated inflammatory response to IAV infection in the absence of OSM (Fig. 1H).

### OSM deficiency results in exacerbated IFN-I responses and lung immunopathology during IAV infection and increased susceptibility to viral mimic challenge.

**Fig 2.**
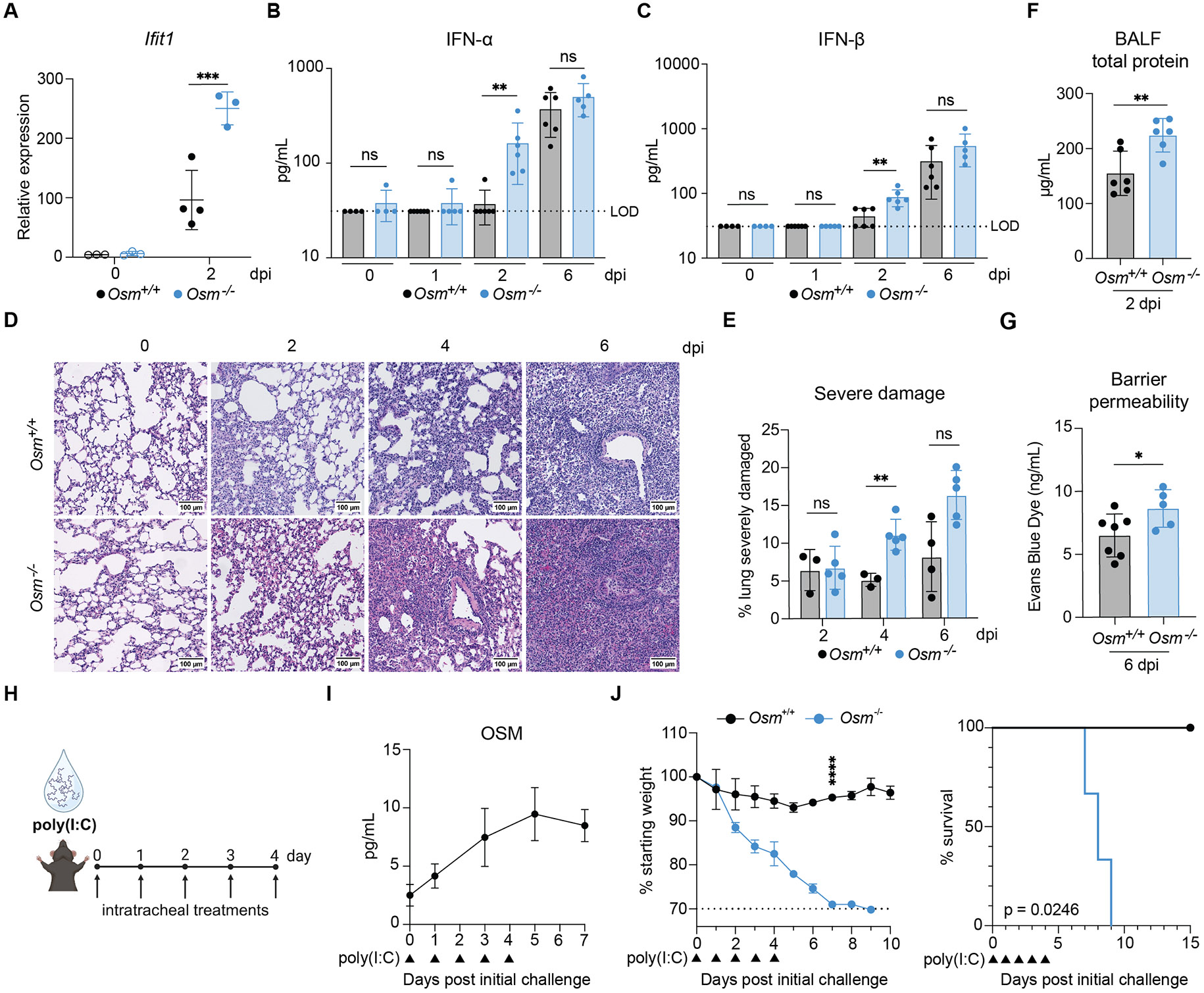
OSM deficiency results in exacerbated IFN-I responses and lung immunopathology during IAV infection and increased susceptibility to viral mimic challenge. **(A** to **C)** Mice were infected i.n. with 225 PFU of A/WSN/1933 (H1N1) or mock-infected with PBS (0 dpi). Whole-lung RNA was collected at indicated time points and *Ifit1* transcript expression measured by RT-qPCR (A) (n = 3 to 4 mice per group). Protein from BALF was collected at indicated time points, and IFN-α (B) and IFN-β (C) protein levels were assessed by ELISA (n = 4 to 6 mice per group). (**D** to **G**) Mice were infected i.n. with 225 to 450 PFU of A/WSN/1933 (H1N1). Representative histological images of mouse lungs at baseline and throughout IAV infection (D). Histological scoring of the percentage of the lung exhibiting severe damage throughout IAV infection (E) (n = 3 to 5 mice per group). BALF was collected at 2 dpi for total protein quantification (F) (n = 4 to 6 mice per group). Lung barrier permeability was assessed with an Evans blue dye assay on BALF at 6 dpi (G) (n = 5 to 7 mice per group); representative of at least two independent experiments. (**H** to **J**) Mice were treated intratracheally (i.t.) with 33.75 to 50 μg of poly(I:C) daily for 5 consecutive days. Schematic of experimental design (H). BALF was collected at indicated time points and OSM protein levels detected by ELISA (I) (n = 4 to 5 mice per group). Mice were monitored for body weight (left panel) and survival (right panel) (J) (n = 3 mice per group); representative of at least two independent experiments. Male mice were used for (G) and (J), and female mice were used for the remaining experiments. Relative expression calculated as (2^−ΔCt^)*1000. In (A to G), symbols represent individual mice, and bars are means. In (I) and (J), symbols represent mean data. In all graphs, error bars indicate SD, *P ≤0.05, ** *P* ≤0.01, *** *P* ≤0.001, **** *P* ≤0.0001, ns = not significant. Two-way ANOVA for (A) and (E); Mann-Whitney U test for (B), (C), and (F); unpaired Student’s t test for (G), two-way ANOVA up to day 7 for (J), left panel; and log-rank Mantel-Cox test for (J) right panel. LOD, level of detection.

As increased morbidity and mortality occurred at later time points after IAV infection, we performed bulk RNA-seq on whole lungs from *Osm*^+/+^ and *Osm*^−/−^ mice at 3 and 7 dpi (fig. S7 and data S3). At 3 dpi, pathway enrichment analyses (22, 23) indicated a more prominent IFN-I signature in *Osm*^−/−^ mice as compared with *Osm*^+/+^ mice (fig. S7). These findings were corroborated by an increase in lung ISG expression, including *Ifit1*, *Isg15*, and *Irf7,* and an increase in IFN-I transcripts, including *Ifnb* and *Ifna4* (Fig. 2A and fig. S8). *Osm*^−/−^ mice also showed elevated IFN-ɑ and IFN-β in BALF at 2 dpi compared with *Osm*^+/+^ mice (Fig. 2, B and C). We investigated histological differences at 2, 4, and 6 dpi (Fig. 2, D and E, and fig. S9, A and B) and found that *Osm*^−/−^ mice displayed more severe damage at 4 dpi (Fig. 2E). IAV-infected *Osm*^−/−^ mice also exhibited increased BALF total protein levels and barrier permeability compared with *Osm*^+/+^ mice (Fig. 2, F and G), indicating a loss of lung barrier integrity, a known hallmark of IFN-mediated immunopathology (2). Elevated immune infiltration was also observed in *Osm*^−/−^ mice, with increased numbers of neutrophils at both 2 and 6 dpi, monocytes at 2 dpi, and interstitial macrophages (IMs)/monocyte-derived macrophages (moMacs)—which are indistinguishable using conventional gating schemes—at 6 dpi (fig. S10 and fig. S11A to C). However, we found no differences in AM, plasmacytoid dendritic cell, or lymphocyte numbers between *Osm*^+/+^ and *Osm*^−/−^ mice at either early- or late-infection time points (fig. S10 and fig. S11, D to J). Notably, we observed no differences in lung damage or IFN-I levels at baseline (Fig. 2, B and C, and figs. S8 and S9C), indicating that although OSM regulated ATII gene expression, this did not lead to overt lung pathology under steady-state conditions.

To evaluate whether the increases in IFN-I and tissue damage correlated with differences in viral load, we performed viral plaque assays on BALF collected from mice at 1, 2, and 6 dpi. We did not observe differences in viral load at 1 or 6 dpi and detected no viremia in either *Osm*^+/+^ or *Osm*^−/−^ mice (fig. S12). However, at 2 dpi, we detected an increase in viral load in *Osm*^−/−^ mice compared with wild type (fig. S12A). Although equivalent viral loads at 6 dpi suggested that viral burden was not the driver of the IAV survival phenotype, we sought to avoid this confounding factor. To do so, we utilized an additional *in vivo* model using intratracheal (i.t.) delivery of poly(I:C) (Fig. 2H), which is a potent inducer of IFN-I (24) and tissue damage (fig. S13A).

We found that OSM levels increased locally in BALF and lung in response to i.t. poly(I:C) treatment, but not systemically in plasma (Fig. 2I and fig. S13, B and C). Furthermore, *Osm*^−/−^ mice succumbed to i.t. poly(I:C) treatment (Fig. 2J), an outcome conserved across sexes (fig. S13D). We detected *Osm* expression throughout the myeloid compartment in sorted AMs, IMs/moMacs, neutrophils, and monocytes from the lungs of wild-type mice at steady state and at 7 days post-initial challenge (dpc) (fig. S13, E and F). Given the localized presence of OSM in the lung after viral stimuli, we investigated whether OSM provided protection in mice treated intraperitoneally (i.p.) with poly(I:C) for 3 consecutive days. *Osm*^+/+^ and *Osm*^−/−^ mice both exhibited high levels of morbidity in response to systemic poly(I:C) (fig. S13G). These results suggested that OSM provided local protection against viral stimuli in the lung but did not confer protection against systemic viral stimuli.

### Macrophage-derived OSM is required for survival during poly(I:C) challenge.

To determine the importance of macrophage-derived OSM upon i.t. poly(I:C) challenge, we developed a macrophage-specific conditional knockout mouse model by crossing *Osm*^*fl/fl*^ and *Fcgr1*^Cre^ (CD64^Cre^) (25) mouse lines. We validated the loss of *Osm* in sorted AMs and IMs/moMacs at 7 dpc (fig. S14A). Additionally, we found CD64 expression to be exclusive to F4/80^+^CD11b^+^Ly6C^+^ cells in the lung and not in the blood, indicating that circulating monocytes do not express CD64 at the protein level (fig. S14B). To confirm that macrophages were the major source of OSM in the context of viral stimuli, we measured lung OSM levels after poly(I:C) challenge and observed diminished RNA and protein expression in OSM-deficient mice (fig. S14, C and D). Macrophage-specific *Osm* deletion also reduced OSM levels during peak production after IAV infection (Fig. 1A and fig. S14E). We assessed myeloid cell phagocytic capacity and baseline numbers in *Fcgr1*^Cre^*Osm*^*fl/fl*^ mice and found no defects in macrophage or neutrophil phagocytosis (fig. S14F and supplementary text 1) and no differences in AM, IM/moMac, or monocyte numbers, with a reduction in neutrophils (fig. S14G). Additionally, we examined *Osmr* expression in various cell types in poly(I:C)-treated and -untreated *Fcgr1*^Cre^*Osm*^*fl/fl*^ and *Osm*^*fl/fl*^ mice and did not observe differences in receptor expression across genotypes (fig. S14H).

**Fig 3.**
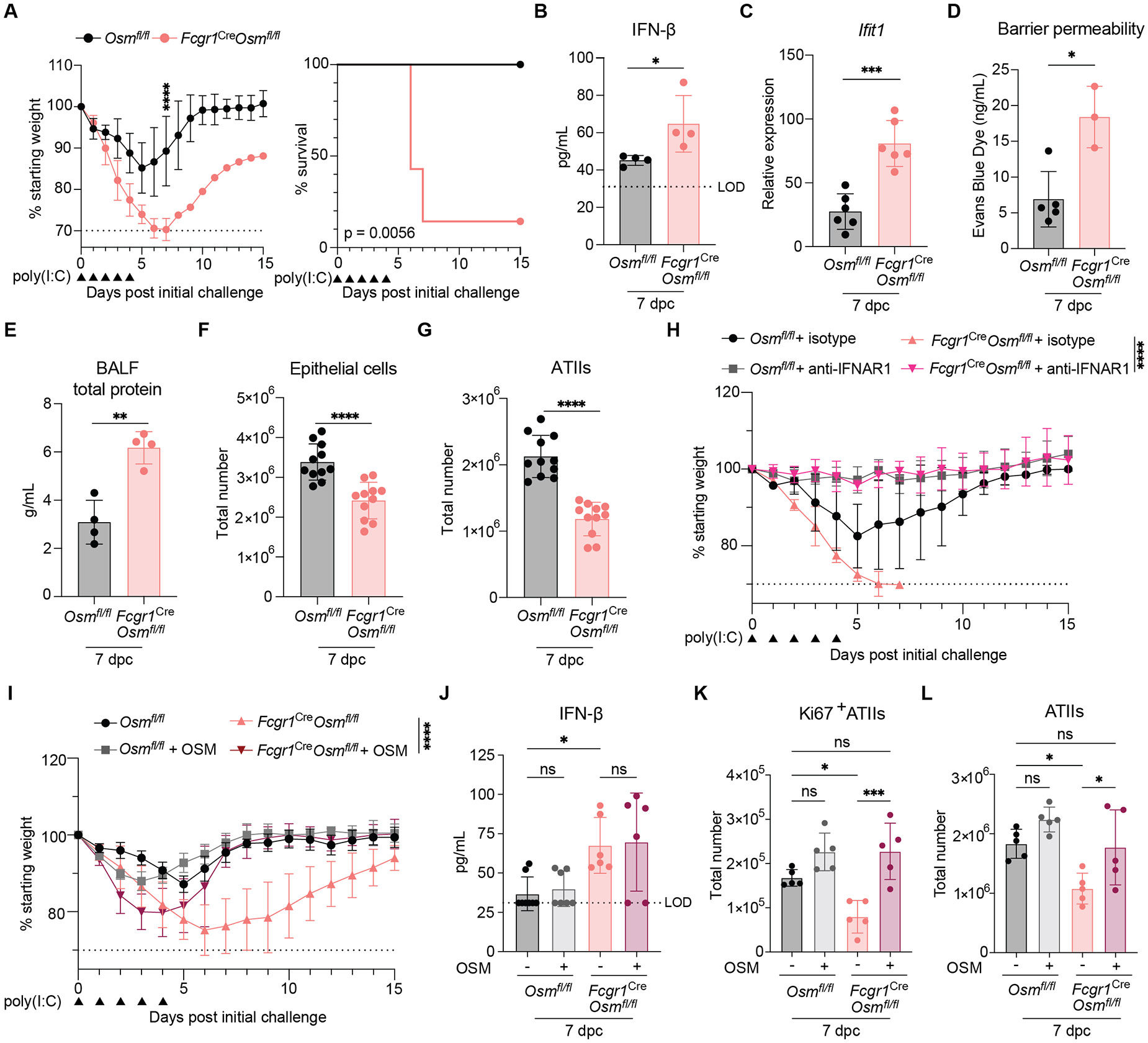
Macrophage-derived OSM is required for survival during poly(I:C) challenge. **(A** to **G)** Mice were treated i.t. with 33.75 to 50 μg of poly(I:C) daily for 5 consecutive days. Mice were monitored for body weight (left panel) and survival (right panel) (A) (n = 5 to 7 mice per group). BALF was collected at 7 days post-initial challenge (dpc), and IFN-β protein levels were detected by ELISA (n = 4 mice per group) (B). Whole-lung RNA was collected at 7 dpc and *Ifit1* transcript expression measured by RT-qPCR (C) (n = 6 mice per group). Representative of at least two independent experiments for (A) to (C). Lung barrier permeability was assessed with an Evans blue dye assay on BALF at 7 dpc (D) (n = 3 to 5 mice per group). BALF was collected at 7 dpc for total protein quantification (n = 4 mice per group) (E). Lungs were collected and total epithelial cell (F) and ATII cell (G) numbers at 7 dpc were determined with flow cytometry (n = 11 mice per group); pooled from three independent experiments. (**H**) Mice were treated i.t. with 33.75 to 50 μg of poly(I:C) daily for 5 consecutive days. In addition, mice were treated intravenously (i.v.) with either 200 μg of anti-mouse IFNAR1 or isotype control antibody diluted in 100 μl PBS on −1, 0, 1, 3, and 4 dpc. Mice were weighed daily (n = 3 to 4 mice per group). (**I** to **L**) Mice were treated i.t. with 1 μg of mouse recombinant (rOSM) in addition to 33.75 to 50 μg of poly(I:C) daily for 5 consecutive days. Mice were weighed daily (I) (n = 4 to 5 mice per group). BALF was collected at 7 dpc and IFN-β protein levels detected by ELISA (J) (n = 6 to 8 mice per group). Lungs were collected and Ki67^+^ ATII (K), and total ATII (L) numbers at 7 dpc were determined with flow cytometry (n = 5 mice per group). Representative of at least two independent experiments for (H) to (L). A combination of female and male mice was used for experiments in this figure. Relative expression calculated as (2^−ΔCt^)*1000. In (A), (H), and (I), symbols represent mean data. In (B) to (G) and in (J) to (L), symbols represent individual mice, and bars are means. In all graphs, error bars indicate SD; * *P* ≤0.05, ** *P* ≤0.01, *** *P* ≤0.001, **** *P* ≤0.0001, ns = not significant. Two-way ANOVA up to day 7 for (A), left panel, up to day 6 for (H), and up to day 15 for (I); log-rank Mantel-Cox test for (A), right panel; Mann-Whitney U test for (B) and (D), unpaired Student’s t test for (C), (E), (F), and (G); nonparametric one-way ANOVA for (J); and one-way ANOVA for (K) and (L). LOD, level of detection.

Macrophage-specific deletion of *Osm* during poly(I:C) challenge led to increased morbidity and mortality (Fig. 3A), increased BALF IFN-I levels and infiltration of IMs/moMacs, monocytes, and neutrophils into the lung, and a decrease in AMs (Fig 3B and fig. S15A). This was accompanied by increased IFN and ISG transcript expression, enhanced barrier permeability, and elevated levels of total protein in BALF (Fig. 3, C to E, and fig. S15B and C).

Previous studies demonstrated IFN-driven disruption of lung epithelial barrier function (2, 24). We tested whether, after challenge, mice lacking macrophage-derived OSM exhibited a loss of epithelial cells, which might explain their compromised barrier integrity. *Fcgr1*^Cre^*Osm*^*fl/fl*^ mice showed a reduction in total lung epithelial cells and specifically ATIIs at 7 dpc (Fig. 3, F and G), suggesting alveolar damage.

To test the hypothesis that OSM protected mice from succumbing to poly(I:C) treatment through an IFN-I-mediated pathway, we administered intravenous (i.v.) IFN-ɑ/β receptor antibodies [anti-interferon α/β receptor subunit 1 (IFNAR1)] to *Fcgr1*^Cre^*Osm*^*fl/fl*^ and *Osm*^*fl/fl*^ mice during treatment with poly(I:C) (fig. S16A). Upon IFNAR1 neutralization, *Fcgr1*^Cre^*Osm*^*fl/fl*^ mice exhibited no differences in morbidity or mortality compared with anti-IFNAR1 treated *Osm*^*fl/fl*^ mice (Fig. 3H, fig. S16A, and supplementary text 2). Moreover, reconstitution of OSM in the respiratory tract by administration of recombinant mouse OSM, lessened morbidity in *Fcgr1*^Cre^*Osm*^*fl/fl*^ mice after poly(I:C) treatment (Fig. 3I and fig. S16B). Local administration of OSM did not change BALF IFN-I levels in *Fcgr1*^Cre^*Osm*^*fl/fl*^ mice after poly(I:C) treatment (Fig. 3J) but led to elevated numbers of Ki67^+^ ATIIs, total ATIIs, and an increase in Ki67^+^ ATII proportions (Fig. 3, K and L, and fig. S16C). This suggested that although IFN-I signaling was necessary for morbidity in the absence of OSM, it was not sufficient; moreover, OSM signaling restored ATII proliferation after epithelial damage.

### OSM administration rescues ATII transcriptional states in *Osm*-deficient mice.

**Fig 4.**
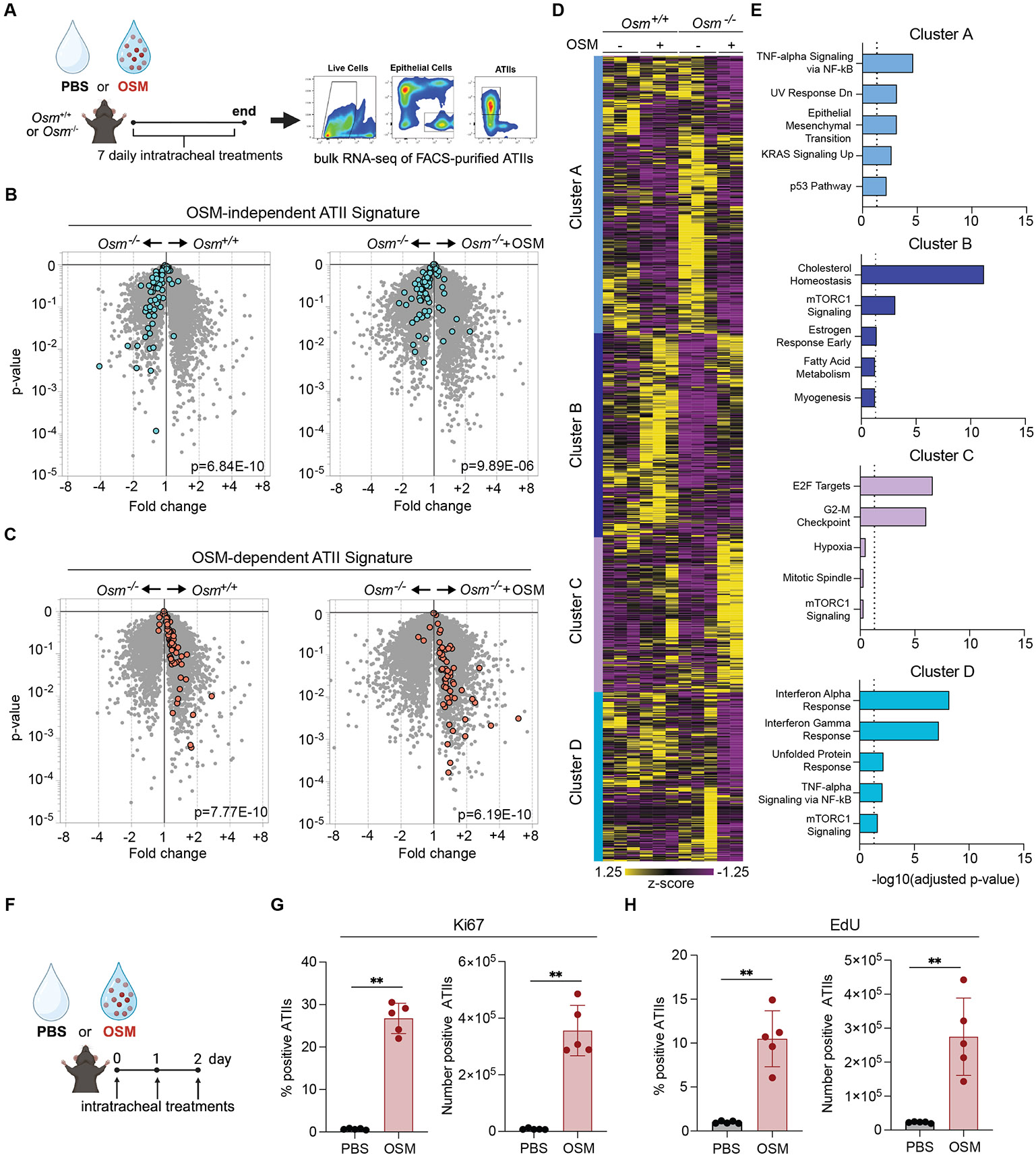
Administration of OSM rescues ATII cell states and induces proliferation. **(A** to **E)** Mice were treated i.t. with either PBS or 1 μg of OSM daily for 7 consecutive days. The following day, lungs were collected, and ATII cells were sorted for bulk RNA-seq analysis. Schematic representation of experimental design (A). Volcano plot for cluster 1 (OSM-independent) ATII gene signature of PBS-treated *Osm*^+/+^ versus PBS-treated *Osm*^−/−^ mice and volcano plot of rOSM-treated *Osm*^−/−^ versus PBS-treated *Osm*^−/−^ mice (B). Volcano plot for cluster 2 (OSM-dependent) ATII gene signature of PBS-treated *Osm*^+/+^ versus PBS-treated *Osm*^−/−^ mice and volcano plot of rOSM-treated *Osm*^−/−^ versus PBS-treated *Osm*^−/−^ mice (C). Heatmap depicting the expression levels of genes differentially expressed between groups (twofold differential) and *k*-means clustered by row. Rows represent genes, and columns represent samples (D). The resulting clusters were analyzed using MSigDB pathway enrichment in Enrichr. The top five enriched pathways for each cluster are displayed. The dotted line represents the significance threshold for the adjusted *P* value, set at *P* < 0.05 (E). [n = 2 to 3 mice per group for (A) to (E)]. (**F** to **H**) Mice received daily i.t. administrations of 1 μg mouse rOSM and i.p. injections of 1 μg EdU for 3 consecutive days. Schematic representation of experimental design (F). Lungs were processed 24 hours after the final treatment, and Ki67+ ATII (G) and EdU+ ATII (H) cell proportions (left panels) and numbers (right panels) were determined with flow cytometry (n = 5 mice per group); representative of at least two independent experiments. Female mice were used for experiments in this figure. In (G) and (H), symbols represent individual mice, bars are means, and error bars indicate SD; ** *P* ≤0.01. Mann-Whitney U test for (G) and (H). TNF-alpha, tumor necrosis factor alpha, UV response Dn, ultraviolet response down-regulation; mTORC1, mammalian target of rapamycin complex 1; KRAS, Kristen rat sarcoma virus.

We hypothesized that in addition to promoting epithelial cell proliferation, local OSM treatment could restore the OSM-dependent ATII cell state (cluster 2) (Fig. 1, G and H). We administered i.t. OSM to *Osm*^+/+^ and *Osm*^−/−^ mice, harvested ATIIs, and performed bulk RNA-seq (Fig. 4A, fig. S17A, and data S4). On the basis of the clusters identified from scRNA-seq data (Fig. 1H), ATII cluster 1 (OSM-independent)-enriched genes were increased in ATIIs from *Osm*^−/−^ mice compared with *Osm*^+/+^ mice, and ATII cluster 2 (OSM-dependent)-enriched genes were increased in *Osm*^+/+^ mice (Fig. 4, B and C). ATII cells from *Osm*^−/−^ mice treated with i.t. OSM, when compared to those from *Osm*^−/−^ mice, recapitulated the differences observed between *Osm*^+/+^ and *Osm*^−/−^ ATIIs, suggesting restored gene expression in response to OSM treatment (Fig. 4, B and C).

We identified all genes that were at least twofold differentially expressed in any pairwise comparison between groups and performed *k*-means clustering based on expression values (Fig. 4D and data S4). Cluster A was associated with nuclear factor κB signaling and cluster D was associated with IFN signaling (Fig. 4E). Cluster B, which was enriched for genes associated with cholesterol homeostasis, showed lower expression in *Osm*^−/−^ mice compared to controls, but increased expression after the addition of OSM (Fig. 4E and fig. S17B). The expression of cluster C, which reflected cell-cycle progression, did not vary between *Osm*^+/+^ and *Osm*^−/−^ mice, but was increased upon administration of OSM in *Osm*^−/−^ mice (Fig. 4E and fig. S17B). These data suggested that at steady-state levels, OSM regulated cholesterol homeostasis, a functional hallmark of mature ATIIs (26), whereas elevated OSM levels induced cell-cycle progression.

### OSM promotes epithelial proliferation by overriding IFN-I-driven growth inhibition.

Informed by our findings that OSM increased the number of total and Ki67+ ATIIs in *Fcgr1*^Cre^*Osm*^*fl/fl*^ mice after poly(I:C) treatment, we investigated whether, even in the absence of damage and OSM deficiency, elevated OSM levels induced ATII proliferation. We administered i.t. OSM to wild-type mice at steady state for 3 consecutive days (Fig. 4F). Mice treated with OSM displayed elevated ATII Ki67 positivity and 5-ethynyl-2′-deoxyuridine (EdU) incorporation compared to with phosphate-buffered saline (PBS)-treated controls (Fig. 4, G and H).

**Fig 5.**
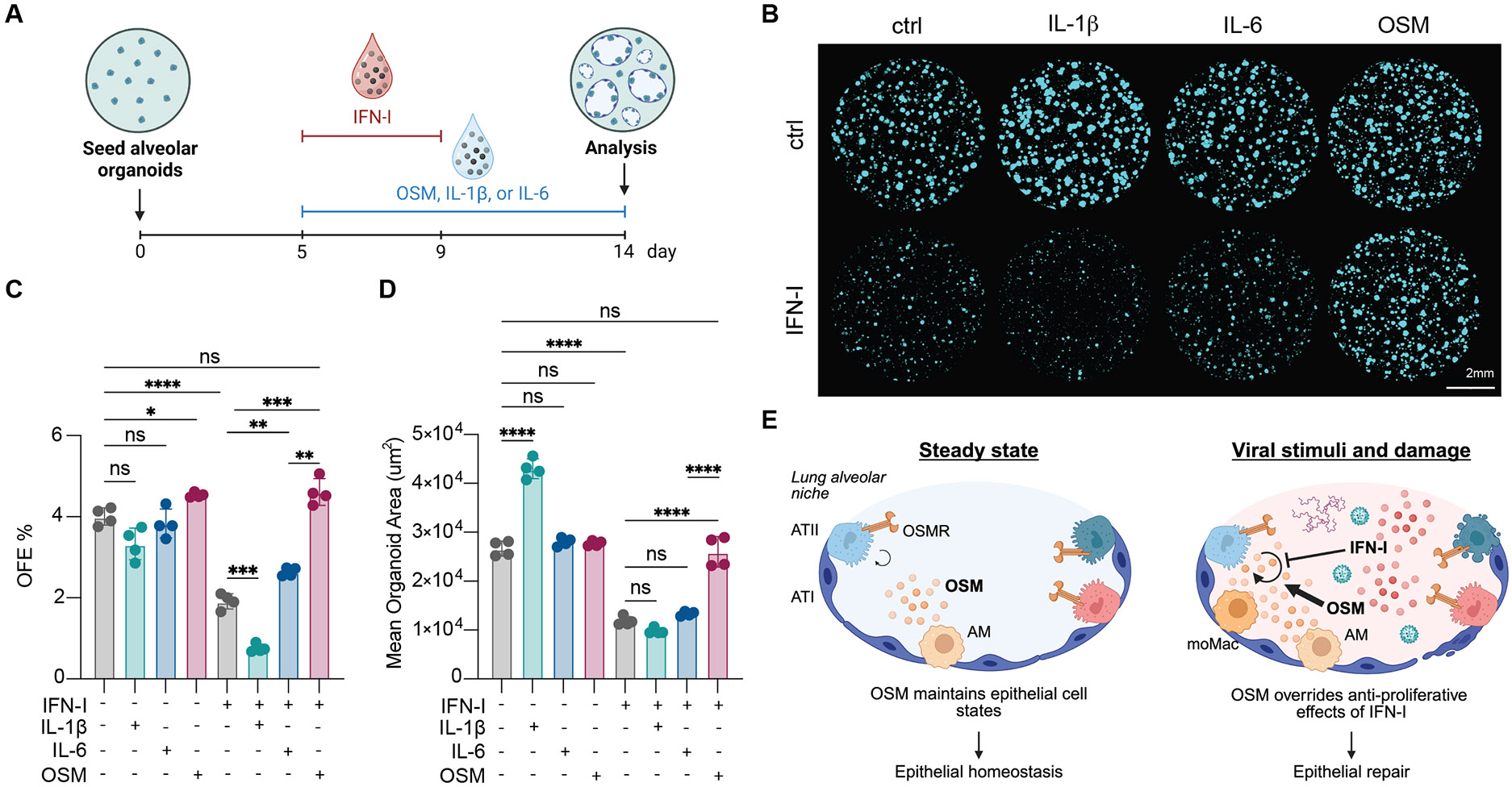
OSM overcomes growth suppressive effects of IFN-I to promote epithelial proliferation. Alveolar organoids were cultured in the presence or absence of IFN-I (200 U/mL; 1:1 mixture of rIFN-α and rIFN-β1) supplemented with mouse rOSM (50 ng/mL), rIL-1β (20 ng/mL), or rIL-6 (50 ng/mL). (**A**) Experimental schematic. Organoids were seeded and allowed to grow for 5 days before treatment. IFN-I treatment began on day 5. Images were taken for analysis 14 days after seeding. (**B**) Representative images of organoid cultures on day 14 after seeding. (**C**) Quantification of organoid forming efficiency (OFE) (n = 4 samples per group). (**D**) Quantification of mean organoid area (n = 4 samples per group). (**E**) Overall model. At steady state, AMs produce OSM which maintains ATII gene expression and function. Upon infection and/or damage of the lung, moMacs and other myeloid cells infiltrate into the alveoli, resulting in elevated OSM levels. This induces proliferation of ATIIs despite ongoing inflammation and antiproliferative IFN-I signaling, restoring the epithelial barrier of the lung. Experiments in this figure are representative of two independent experiments. In (C) and (D), symbols represent individual samples, bars are means and error bars indicate SD; * *P* ≤0.05, ** *P* ≤0.01, *** *P* ≤0.001, **** *P* ≤0.0001, ns = not significant, Brown-Forsythe and Welch ANOVA for (C) and ordinary one-way ANOVA for (D).

We next hypothesized that OSM may induce epithelial proliferation under inflammatory conditions. We grew alveolar organoids (27, 28) in the presence or absence of IFN-I supplemented with interleukin-1β (IL-1β) or STAT3 activators, OSM or IL-6 (Fig. 5A and fig. S17C). IL-1β has been found to promote both epithelial proliferation and differentiation of ATII cells into ATI cells (29, 30). As previously reported (29-31), IFN-I limited organoid forming efficiency (OFE) and mean organoid area, whereas IL-1β increased the area of organoids (Fig. 5, B to D). IL-6 had no effect on either metric. IL-1β was unable to overcome the growth suppressive effects of IFN-I, and IL-6 showed a marginal effect on OFE. By contrast, OSM modestly increased OFE at baseline and completely restored both organoid size and OFE under IFN-I-mediated growth suppression to the levels observed in untreated organoids (Fig. 5, B to D). These data revealed that the growth-promoting function of OSM was dominant over IFN-I. Collectively, this study demonstrated that OSM regulated transcriptional programs in epithelial cells at steady state and promoted host survival by driving ATII proliferation after IFN-I signaling (Fig. 5E).

## Discussion

Our study establishes that macrophage production of OSM is critical for maintaining and restoring lung epithelial homeostasis. Studies have shown that the protective or pathological roles of interferons depend on their temporal expression and spatial activity within the respiratory tract (32, 33). We conclude that OSM mitigates the pathological effects of IFN by promoting epithelial proliferation, even in conditions typically associated with growth suppression. This underscores the integral role of epithelial-immune communication in tissue maintenance and repair (34), which is akin to the role of other macrophage-stromal circuits identified in our previous work (35).

Prior research has described both pro- and anti-inflammatory roles for OSM (36-38). Our findings demonstrate that OSM deficiency alters the transcriptional profiles of epithelial cells, potentially explaining these pleiotropic effects. The regulation of lung epithelial cell states by OSM may also explain previous reports that OSM overexpression in the lung exacerbates inflammation and disease (13, 14). Notably, several studies have shown that increased OSM signaling enhances allergic inflammation (38-42). Our work hints that these prior observations could be due to disruptions in epithelial homeostasis.

Recent studies of OSM across various tissues highlight its emerging role in regulating stem and progenitor cell states (43-47). In muscle (44) and hair follicles (43), OSM was found to regulate stem cell quiescence. OSM was also found to inhibit adipocyte differentiation from mouse embryonic fibroblasts (45) and mesenchymal stromal cells in adipose tissue (46), demonstrating a role in regulating cell differentiation. OSM has also been implicated in tissue repair (48, 49), perhaps through effects on cell differentiation and proliferation, although these mechanisms were not specifically addressed. Our findings extend this theme, identifying an OSM-dependent ATII cell state in the lung and supporting a role for OSM in cell proliferation and/or differentiation. Future investigations will define the precise role of OSM on stem and progenitor cell fate in the respiratory tract and other mucosal tissues.

In the lung, epithelial repair operates concurrently with a robust antiviral response to prevent prolonged disruption of the epithelial barrier and vulnerability to secondary infections. Our study identifies a mechanism of disease tolerance in which macrophage-derived OSM plays a crucial role in stimulating the proliferation of lung epithelial cells, even under the antiproliferative effects of IFN-I. Furthermore, our work suggests that OSM may function as a “counter-inflammatory” signal (50), opposing the harmful effects of inflammation on target tissues rather than simply reducing the overall inflammatory response. These findings also highlight the potential of OSM as a therapeutic agent to activate repair pathways, particularly in severe infections in which expedient restoration of the epithelial barrier is critical.

## Supplementary Material

Data file S1

Data file S2

Data file S4

Data file S3

Supplementary materials

Materials and Methods

Supplementary Text 1 and 2

Figures S1 to S17

Tables S1 and S2

Data Files S1 to S4

References 52 to 58
