# Supplementary material for: Macrophage-derived oncostatin M repairs the lung epithelial barrier during inflammatory damage
Source: Science. Author manuscript; Available in PMC 2025 Oct 23. (PMC12541708; doi:10.1126/science.adi8828)
Supplement: Supplementary materials [file NIHMS2110192-supplement-Supplementary_materials.pdf]

Science
AAAS

Supplementary Materials for

Macrophage-derived oncostatin M repairs the lung epithelial barrier during inflammatory damage

Authors: Daisy A. Hoagland[1,2]†, Patricia Rodríguez-Morales[1,2]†, Alexander O. Mann[1,2], Alan Y. Baez Vazquez[1,2], Shuang Yu[3], Alicia Lai[1,2], Harry Kane[2], Susanna M. Dang[4,5], Yunkang Lin[1,2], Louison Thorens[6], Shahinoor Begum[1,2], Martha A. Castro[1,2], Scott D. Pope[3], Jaechul Lim[3], Shun Li[7], Xian Zhang[7], Ming O. Li[7], Carla F. Kim[4,5,8], Ruaidhrí Jackson[2], Ruslan Medzhitov[3,9,10]*, Ruth A. Franklin[1,2,8]*

Corresponding author: Ruth A. Franklin ruthfranklin@fas.harvard.edu;
Ruslan Medzhitov ruslan.medzhitov@yale.edu

The PDF file includes:

Materials and Methods
Supplementary Text
Figures S1 to S17
Tables S1 and S2
Data Files S1 to S4
References 52 to 58

Materials and Methods

Mice

All animal experiments were performed in accordance with institutional regulations after protocol review and approval by the Institutional Animal Care and Use Committee (IACUC) at Harvard Medical School (protocol IS00003152). Mice were bred and housed in a specific pathogen-free facility, with room temperature set to 71°F (+/-3°), humidity set to 50% (+/-15%), 12:12-hour light/dark cycle, and *ad libitum* access to food (LabDiet 5053) and water. C57BL/6J mice (RRID:IMSR_JAX:000664) and DsRed.T3 (RRID:IMSR_JAX:006051) were obtained from Jackson Laboratories (JAX), *Fcgr1*^{Cre} mice were provided by Dr. Ming Li (MSKCC) (*25*), and gene targeted *Osm* "Knockout-first" mice were obtained from the KOMP repository (RRID:MMRRC_059829-UCD). To generate conditional *Osm* alleles, mice were bred to the FLPo deleter strain (RRID:IMSR_JAX:012930). *Osm* conditional mice were then bred to *Fcgr1*^{Cre} mice to achieve conditional deletion. Animals were euthanized by administration of a lethal dose of ketamine/xylazine mixture. Female and male mice aged 8 to 12 weeks were used for all experiments, as specified.

Infection and *in vivo* treatments

Mice were acclimated to the facility for at least one week before experimental challenge. For influenza virus or mock infection, mice were anesthetized with a ketamine/xylazine mixture, then administered 225 - 450 plaque forming units (PFU) of virus or PBS (phosphate-buffered saline) intranasally (i.n.) in a 30 µL volume. For intratracheal (i.t.) treatments, mice were anesthetized by inhalation of isoflurane/propanediol and administered 33.75 - 50 µg of high molecular weight poly(I:C) (InvivoGen) diluted in PBS to 50 µL, or PBS alone, using a pipette. For IFNAR1-blockade treatments, mice were injected intravenously (i.v.) with 200 µg of *InVivo*Plus anti-mouse IFNAR1 or rat IgG2a isotype control (Bio X Cell) in 100 µL volume. For OSM rescue experiments in the context of poly(I:C) challenge, mice were administered 1 µg of recombinant mouse OSM (rOSM; Bio-Techne) or PBS in addition to daily treatment with 33.75 - 50 µg of poly(I:C). For systemic poly(I:C) challenge, mice were treated intraperitoneally (i.p.) with 100 µg of poly(I:C) daily for three consecutive days. For steady state rOSM rescue experiments, mice received daily i.t. administrations of 1 µg of rOSM or PBS in a 50 µL volume for seven consecutive days. For phagocytosis assays, mice were i.t. administered 50 µg pHrodo E.Coli BioParticles (Thermo Fisher Scientific) 3 hours before euthanasia. For steady state lung epithelium proliferation assays, mice received daily i.t. administrations of 1 µg rOSM or PBS in a 50 µL volume and i.p. injections of 1 µg EdU (Thermo Fisher Scientific) in a 200 µL volume for three consecutive days. Mice were euthanized and tissues collected 24 hours after the final treatment. For the Evans Blue Dye (EBD) lung barrier permeability assay, mice were injected i.p. with 10 mg/kg of EBD (Sigma) 16 hours before euthanasia.

Plaque assay and virus propagation

Influenza virus strain A/WSN/33 was originally obtained from Dr. Peter Cresswell (Yale University School of Medicine). The virus was propagated and titered by plaque assay using Madin-Darby Canine Kidney (MDCK) cells (ATCC, CCL-34, NBL-2). To determine viral titer, a 10-fold serial dilution of virus was prepared in 0.1% BSA in PBS in 200 µL volume. The cells were infected at 37°C for 1 hour and shaken every 15 minutes. Cells were washed twice with PBS and an agarose overlay of 1% agarose gel mixture including TPCK Trypsin (1:2000), 0.21% BSA,

0.225% $NaHCO_3$, 100 U/mL penicillin/streptomycin in MEM was added to each well and the plate was incubated at 37°C for 48-72 hours upside down. Wells were fixed with 10% formalin for at least two hours. Gels were removed, 1 mL of crystal violet solution was added to each well, and the plate was incubated at room temperature for 30 minutes. For viral propagation, MDCK cells were infected at an MOI of 0.002 in 100 µL of DMEM + 1% BSA per well and incubated for 10 minutes at 37°C. 1.5 mL of DMEM + 1% BSA were added to each well and cells were left to grow for 48 hours. Cells were spun down at 850G for 10 minutes and supernatant collected.

Lung isolation for flow cytometry and cell sorting

Lungs were harvested and processed as previously described (*52*). Briefly, mice were euthanized and perfused with 2 mM EDTA in PBS. For flow cytometry analysis of myeloid and epithelial cells, the trachea was exposed, nicked, and a 22-gauge catheter was inserted. A 1 mL-PBS-filled syringe was attached to the catheter and used to inflate then deflate lungs to collect cells present in BALF. The syringe was replaced with a 1 mL-dispase-filled syringe and lungs were inflated. Inflated lungs were removed, and lung lobes were chopped and immersed in enzyme solution (100 µg/mL DNase and 83 µg/mL Liberase in RPMI) and placed in a 37°C shaker for 40 minutes. BALF and lung digest cells were filtered through a 70 µm cell strainer to obtain a single cell suspension and exposed to hypotonic lysis (ACK lysing buffer, ThermoFisher) to remove red blood cells. For flow cytometry analysis of lymphocytes and pDCs, lungs were removed, and lung lobes were chopped and immersed in enzyme solution (100 µg/mL DNase and 2 mg/mL Collagenase IV (Worthington Biochemical) in PBS). Lungs were placed in a 37°C shaker for 30 minutes. Digested cells were filtered through a 70 µm cell strainer to obtain a single cell suspension and exposed to hypotonic lysis to remove red blood cells. Antibodies used for flow cytometry are listed in table S1. Cell viability was determined using Zombie Aqua Fixable Viability Kit (BioLegend) following the manufacturer's protocol for flow cytometry and DAPI at 10.9 µM (BioLegend) for cell sorting. Samples were Fc-blocked with anti-mouse CD16/32 antibodies (1:250). For surface staining, cells were fixed in 2% PFA for 20 minutes at room temperature, then washed. For intracellular staining of Ki67 or Foxp3, following surface staining and washes, cells were fixed and permeabilized overnight using Foxp3 Fix/Perm (eBioscience), and staining was performed for 1 hour at room temperature. Analysis of EdU uptake by flow cytometry was performed using the Click-iT® Plus EdU assay kit (ThermoFisher) following manufacturer instructions. To calculate cell numbers, 123count eBeads (ThermoFisher) were used and calculations were performed per manufacturer instructions. For pSTAT3 staining, following surface staining and fixation, samples were washed and permeabilized overnight in 90% methanol at -20°C. After permeabilization, samples were washed with ice-cold FACS buffer (1% FBS in PBS) and stained for pSTAT3 together with antibodies conjugated to methanol-sensitive fluorophores. Flow cytometry samples were acquired on a Symphony A5 or A1 (BD) and analyzed using FlowJo (Tree Star) software. Samples for sorting were acquired using an Aria 561 (BD) or MoFLo Astrios (Beckman Coulter Life Sciences).

Collection and analysis of blood, whole lung, and BALF

Blood was collected and plasma was isolated using lithium heparin coated plasma separator tubes (BD). To collect BALF, following euthanasia, the trachea was exposed, nicked, and a 22-gauge catheter was inserted. A 1 mL-PBS-filled syringe was attached to the catheter and used to inflate and deflate the lungs twice. The lungs were collected into PBS-filled microtubes with ceramic beads and homogenized using a Bead Mill Homogenizer (OMNI International). The homogenized lungs were centrifuged at 10,000G for 10 minutes at 4°C. The BALF was centrifuged at 300G for

5 minutes at 4°C. Supernatants were aliquoted and frozen down at -80°C for protein analysis. Total BALF protein levels were quantified using the Bio-Rad Protein Assay. Mouse IFN-α2 was quantified using the LumiKine Xpress mIFN-α 2.0 kit (Invivogen) and mouse IFN-β using the LumiKine Xpress mIFN-β 2.0 kit (Invivogen) according to manufacturer instructions. Mouse OSM was quantified using the Mouse OSM Quantikine ELISA kit (R&D Systems) according to manufacturer instructions. For EBD barrier permeability assay, 200 μL of collected BALF was read at 620 nm absorbance in a 96-well plate. All plates were read using a BioTek Synergy HTX plate reader. For flow cytometry analysis of circulating monocytes, blood was collected into 2 mM EDTA, and red blood cells were removed using ACK lysing buffer.

Histological analysis

For histological analysis, following euthanasia, mice were perfused with 2 mM EDTA in PBS and lung lobes were fixed in 10% formalin for 48 hours, then embedded, sectioned, and H&E stained by the HMS Rodent Histopathology core. Histology images were acquired using an Olympus VS200 Slide scanner with a color camera, using the UPlan X Apo 10x/0.4 Air objective. Severe damage quantification was performed using QuPath software. Representative training regions were selected and annotated for background, healthy tissue, and severe damage. These regions were utilized to train the pixel classifier, which was subsequently optimized and applied to all slides to obtain classification areas. Severe damage was defined as regions characterized by thickened alveolar walls and immune infiltrates within the alveolar walls and alveoli. The percentage of lung severely damaged was calculated as the area classified as severe damage divided by the total lung area, normalizing for background differences. Representative images were obtained using ImageJ software.

Immunofluorescence staining and imaging

Following euthanasia, mice were perfused with 2 mM EDTA in PBS and the trachea was exposed, nicked, and a 22-gauge catheter was inserted. A 1 mL syringe filled with a 50% OCT and 50% PBS + 4% PFA solution was attached to the catheter and used to inflate the lungs. Inflated lungs were removed and fixed in 2% PFA on ice for 2 hours. Fixed lungs were incubated in 30% sucrose at 4°C for 24 hours, followed by an additional 24-hour incubation in 15% sucrose and 50% OCT under the same conditions. Lungs were embedded in OCT and cryosectioned at 14 μm and mounted on slides. Sectioned samples were blocked in 3% BSA and 10% donkey serum with 0.1% Triton-X in PBS for 1 hour at room temperature. Sections were incubated overnight at 4°C with primary antibodies (rabbit anti-pro-SP-C, 1:1000, Abcam and rabbit anti-uteroglobin, 1:200, Abcam) diluted in blocking solution. Sections were washed with 0.1% Triton-X in PBS and stained with secondary antibody (donkey anti-rabbit AF647, 1:200, Biolegend) for 1 hour at room temperature. After washing, sections were stained with DAPI (1:10,000) for 5 minutes at room temperature, washed again, and mounted with Diamond Antifade (ThermoFisher). Images were taken on an Olympus VS200 Slide scanner equipped with a Hamamatsu Orca fusion BTsCMOS monochrome camera, using the UPlan X Apo 10x/0.4 Air objective. Representative images were obtained using ImageJ software.

RNA extraction and quantification

Tissue samples were collected into TRIzol reagent (Invitrogen) and homogenized in microtubes with ceramic beads and RNA was extracted using the Direct-zol RNA Miniprep kit (Zymo Research). RNA from *in vitro* samples was extracted using phenol-chloroform isolation and co-

precipitated with GlycoBlue (Invitrogen). For sorted cells, samples were collected into TRIzol LS reagent (Invitrogen) and RNA was harvested via phenol-chloroform isolation and co-precipitated with GlycoBlue. RNA samples used for *Ifnb, Ifna4*, and *Ifnl2* RT-qPCR analysis were treated with DNA-free DNA Removal Kit (Invitrogen) before cDNA synthesis. cDNA synthesis was performed using MMLV reverse transcriptase (Takara Bio) and oligo(dT) primers. RT-qPCR reactions were performed on the Applied Biosystems QuantStudio5 Real-Time PCR System (ThermoFisher) using PowerUp SYBR Green Master Mix (ThermoFisher). Relative expression in RT-qPCR analysis was calculated as $(2^{-\Delta Ct})*1000$. ΔCt was calculated by subtracting *Rpl13* Ct values from Ct values of genes of interest. Values displayed as -Ct were normalized by RNA input. All oligonucleotides are listed in table S2.

<u>Cell culture</u>

To generate bone marrow-derived macrophages (BMDMs), female C57BL/6J mice were euthanized and their femurs and tibias were isolated and cleansed with ethanol. Bone marrow was flushed with RPMI 1640 and cells were treated with ACK lysis buffer. Cells were cultured in complete RPMI (cRPMI) (RPMI 1640 + 2 mM L-glutamine, 1 mM sodium pyruvate, 10 mM HEPES, 200 U/mL penicillin/streptomycin, 10% FBS, and 2-mercaptoethanol (BME)). The following day (day 1), non-adherent cells were harvested, and 10 x 10^6 cells were resuspended in a mixture of 70% cRPMI and 30% L929-conditioned media and plated on a petri-dish. Additional 70% cRPMI and 30% L929-conditioned media was added on day 4. Cells were used for experiments on day 6. All cell cultures were maintained in a 37°C incubator at 5% CO_2. Treatment conditions were as follows: ER stress was induced by 5 μM thapsigargin, heat shock performed at 42°C, and cells stimulated with 10 ng/mL LPS or 50 μg/mL poly(I:C). MDCK cells were cultured in complete DMEM (2 mM L-glutamine, 1 mM sodium pyruvate, 10 mM HEPES, 200 U/mL penicillin/streptomycin, and 10% FBS). For AM stimulation experiments, cells collected from bronchoalveolar lavage of female C57BL/6J mice were plated in tissue culture-treated 24-well plates and cultured in cRPMI under the following conditions: 50 μg/mL poly(I:C), 100 ng/mL GM-CSF (Bio-Techne), or a combination of both. For moAM stimulation experiments, lungs from female C57BL/6J mice infected with IAV for 28 days were collected. Cells were isolated, sorted, plated in tissue culture-treated 96-well plates, and cultured in cRPMI with stimulation using a combination of 50 μg/mL poly(I:C) and 100 ng/mL GM-CSF. After 24 hours, the supernatants were collected and frozen for subsequent protein analysis.

<u>Organoid culture</u>

DsRed.T3 mice were anesthetized with avertin, perfused with 10 mL PBS, and intratracheally instilled with 2 mL dispase (Corning). Lungs were minced and incubated in enzyme solution (0.0025% DNase and 100 mg/mL Collagenase/Dispase (Roche) in PBS) for 45 minutes at 37°C. Digested cells were filtered through 100 μm and 40 μm cell strainers, and centrifuged for 5 minutes at 4°C and a speed of 215G. Cells were resuspended in red blood cell lysis buffer (0.15 M NH4Cl, 10mM KHCO3, 0.1 mM EDTA) for 1.5 minutes, washed with Advanced DMEM, and resuspended in PF10 (10% FBS in PBS) at 1 million cells/100 μL. Cells were stained for 15 minutes on ice before cell sorting. FACS isolated mouse lung CD31- CD45- EpCAM+ SCA1- viable cells were resuspended in 3D media (DMEM/F12 supplemented with 10% FBS, penicillin/streptomycin, 1 mM HEPES, and insulin/transferrin/selenium (Corning)) at a concentration of 5,000 live cells (trypan blue negative) per 50 μL. As supporting cells, a mix of neonatal stromal cells was isolated as described elsewhere (*27, 28*). The stromal cells were pelleted

and resuspended in growth factor reduced (GFR) Matrigel (Corning) at a concentration of 50,000 cells per 50 µL. Equal volumes of cells in 3D media and supporting cells in GFR Matrigel were mixed and 100 µL were pipetted into a Transwell (Corning). Plates were incubated for 20 minutes at 37°C, 5% CO2 until Matrigel solidified. Finally, 500 µL of 3D media was added to the bottom of the well. 3D media was changed every other day.

Organoid treatment, imaging, and analysis

Organoid cultures were treated with mouse rOSM (50 ng/mL, Bio-Techne), rIL-1β (20 ng/mL, Bio-Techne), or rIL-6 (50 ng/mL, Bio-Techne or PeproTech), either alone or in combination with IFN-I (200 U/mL, a 1:1 mixture of rIFN-α and rIFN-β1, BioLegend). Organoid media were replenished every 2 - 3 days, with IFN-I treatment spanning a total of four days. Plates were imaged at 14 days post-seeding. Images were captured using an In Cell Analyzer 6000 with a sCMOS monochrome camera from PCO with rolling shutter, using a Plan Apo 4X objective with a 0.20 aperture. 488 laser and 605/52 filter used to capture dsRed organoids. Images captured as max intensity projection of 57 Z slices. Images were stitched together and enhanced using non-local mean filtering, followed by threshold binarization and morphological closing of the binary image. Organoids were counted by standard image segmentation. Organoids of area under 5000 μm^2 (corresponding to 80 µm diameter) were excluded from analysis. All analysis was done using in-lab MATLAB scripts. The MATLAB code developed for this study is available at https://github.com/louisonthorens/organoidDetection under a Creative Commons Attribution 4.0 International License (CC-BY 4.0) (*51*).

Bulk RNA sequencing

For tissue samples, RNA was extracted as previously described. For individual cell populations, cells were sorted and RNA extracted using the Direct-zol RNA Miniprep kit. Purified RNA samples were diluted to 2 ng/µL, and 1 µL of the sample was resuspended in 5 µL of TCL buffer with 1% BME. Data was processed for sequencing and normalization according to the Immgen protocol (https://www.immgen.org/img/Protocols/ImmGenULI_RNAseq_methods.pdf). Whole lung bulk RNA sequencing data are available from the NCBI Gene Expression Omnibus (GEO) under accession number GSE249668 and sorted ATII bulk RNA sequencing data under GSE287742.

Transcriptional analysis

Normalized reads were quality filtered based on minimum expression and coefficient of variation and then visualized with volcano plots and FC/FC plots using Multiplot Studio (GenePattern; Broad Institute). Pathway analysis was performed using Enrichr (*23*), incorporating data from the MsigDB (*22*) and Reactome (*53*) databases. Heat map visualizations and clustering were performed using Morpheus (Broad Institute, https://software.broadinstitute.org/morpheus).

scRNA sequencing

Female mice were infected or mock-infected as described above. Two days post-infection, lungs were collected and digested using the dispase-based digest as described above. Hashing was performed by staining eight percent of the whole lung single cell suspension with TotalSeq-B0301 (Biolegend, Catalog #155831) or TotalSeq-B0302 (Biolegend, Catalog #155833) before encapsulation.

Upstream processing of 10x single cell data

Reference files for alignment and counting of 10x single cell reads were prepared using Gencode Release M33 (GRCm39; https://www.gencodegenes.org/) according to build notes provided by 10x genomics (https://www.10xgenomics.com/support/software/cell-ranger/downloads/cr-ref-build-steps) (*54*). Prior to running cellranger mkref, complete H1N1 segment cRNA sequences from LC333182, LC333183, LC333184, LC333185, LC333186, LC333187, LC333188, and LC333189 (https://www.ncbi.nlm.nih.gov/nuccore/) were added to the primary assembly GRCm39 fasta file. Matching GTF file entries were also prepared for H1N1 segment reference files and added to the filtered GRCm39 GTF file. Modified GRCm39 reference files containing H1N1 segment information were then passed to cellranger mkref to complete the 10x genomics reference generation pipeline. Gzipped FASTQ files containing gene and antibody hashtag data were aligned to the H1N1 modified GRCm39 reference files using 10x Genomics Cell Ranger v8.0.1.

Downstream processing of 10x single cell data

Data were analyzed using the Seurat package in R (*55, 56*). Filtered feature matrix.mtx, barcode.tsv and feature.tsv files generated by Cell Ranger were loaded into R using Seurat ≤ v5.1.0. The random seed was set to 9999 using the R set.seed function prior to starting downstream analyses of 10x data. Antibody hashtag reads were normalized using centered log ratio transformation. Hashtag data was demultiplexed using the Seurat HTODemux function with a positive.quantile setting of 0.9. Non-singlet cells were removed from the analysis. Dead and apoptotic cells were filtered based on mitochondrial RNA enrichment using the SeuratWrappers v0.3.5 and flexmix v.2.3-19 RunMiQC function with posterior.cutoff = 0.75, model.slot = "flexmix_model" settings. Cells were further filtered to remove low quality cells and doublets by excluding cells expressing ≤ 500 unique genes and ≥ 9500 unique molecular identifiers (UMIs) and using scDbleFinder v1.18, respectively. Filtered cells were normalized for dimensionality reduction and clustering using the Seurat SCTransform function (*57*). Principal component analysis (PCA) was run using the Seurat RunPCA with n = 100 npcs, and the final uniform manifold approximation (UMAP) was generated using n = 20 dimensions of the previously calculated PCA reduction as input. Nearest neighbor graph construction and cell clustering were applied using the Seurat FindNeighbours with dims = 1:20 and the FindClusters functions with resolution = 0.5, algorithm = 1. Resulting clusters were then manually collapsed to segment broad cellular populations. Log-normalized gene counts for differential expression analysis were produced using the Seurat NormalizeData function using the settings normalization.method = "LogNormalize" and scale.factor = 10000. The following additional R packages were used for data analysis: Seurat v5.1.0, dplyr ≤ v1.1.4, and Nebulosa v1.14.0. ATII subclustering was performed via Seurat via using subset and reprocessed as described above except for resolution = 0.4 for FindClusters and the genotype-dependent subclusters were collapsed. Subcluster gene modules were generated using FindAllMarkers and taking the top 100 genes with an adjusted p-value < 1e-5 from just WT mice. Subcluster gene module enrichment was generated using AddModuleScore and displayed using plotdensity from Nebulosa. All analyses were run on a Pop!_OS 22.04 LTS GNU Linux 64-bit system using Anacoda Python version 24.9.2, R version 4.4.2 (2024-10-31) and RStudio 2024.09.1 Build 394 or a MacBook Pro Apple M3 Max Sonoma 14.5 36GB system and RStudio 2024.09.1 Build 394. The scRNA-seq data generated in this study is available in the Gene Expression Omnibus (GEO) under accession number GSE291698.

Reanalysis of human scRNA-seq data

Data and metadata obtained from published analyses of human lung samples from COVID-19 patients (*19, 20*) were obtained from the Broad Institute Single Cell Portal. Original UMAP dimensions and cell type annotation from deposited data were used for visualization. Plots reflecting these UMAP coordinates, gene expression levels, and cell annotations were generated using the R package ggplot2.

Data analysis and statistics

Statistical tests were performed using GraphPad Prism, with significance defined as $p \leq 0.05$. The log-rank Mantel-Cox test was used to compare survival curves between groups. Two-way ANOVA was conducted to examine the combined and individual impact of two independent variables in a single experimental cohort. One-way ANOVA was applied to compare the means across more than two independent groups, and a non-parametric one-way Welch's ANOVA was used when data did not meet normality or equal variance assumptions. To perform *post hoc* comparison of selected group means we performed Tukey's test on parametric ANOVA data sets and Dunnett's test on non-parametric ANOVA datasets. For-two group comparisons, an unpaired Student's *t* test was used when assumptions of normality and equal variance were met; otherwise, a Mann-Whitney U test was applied. If all control values were identical, a one sample *t* and Wilcoxon test was used. A chi-squared test was used to determine whether gene signatures were up- or down-regulated between a pairwise comparison in volcano plots.

Supplementary Text

Supplementary text 1

ATII-derived GM-CSF is required for AM maintenance in the lung (*10, 16, 17*). Given our findings that OSM is necessary for maintaining ATII cell states, we sought to determine whether perturbations in the ATII population at baseline in *Fcgr1*^{Cre}*Osm*$^{fl/fl}$ mice might indirectly influence the primary function of macrophages, phagocytosis. We administered *E. coli* pHrodo beads i.t. to *Osm*$^{fl/fl}$ and *Fcgr1*^{Cre}*Osm*$^{fl/fl}$ mice and did not detect any differences in phagocytic capacity in macrophages or neutrophils (fig. S14F).

Supplementary text 2

While studies have suggested that IFN-α and IFN-β exhibit different kinetics and functions (*58*), we did not observe different kinetics between these interferons in our influenza time course (Fig. 2B-C). We used IFNAR1 blockade—which inhibits both IFN-α and IFN-β signaling—and observed decreased morbidity and mortality in *Fcgr1*^{Cre}*Osm*$^{fl/fl}$ mice (Fig. 3H, fig. S16A). It remains possible that there are different roles for IFN-α and IFN-β in mediating the increased damage and immune infiltration in poly(I:C)-induced damage in the absence of OSM, however this experimental system cannot discriminate between these interferons.

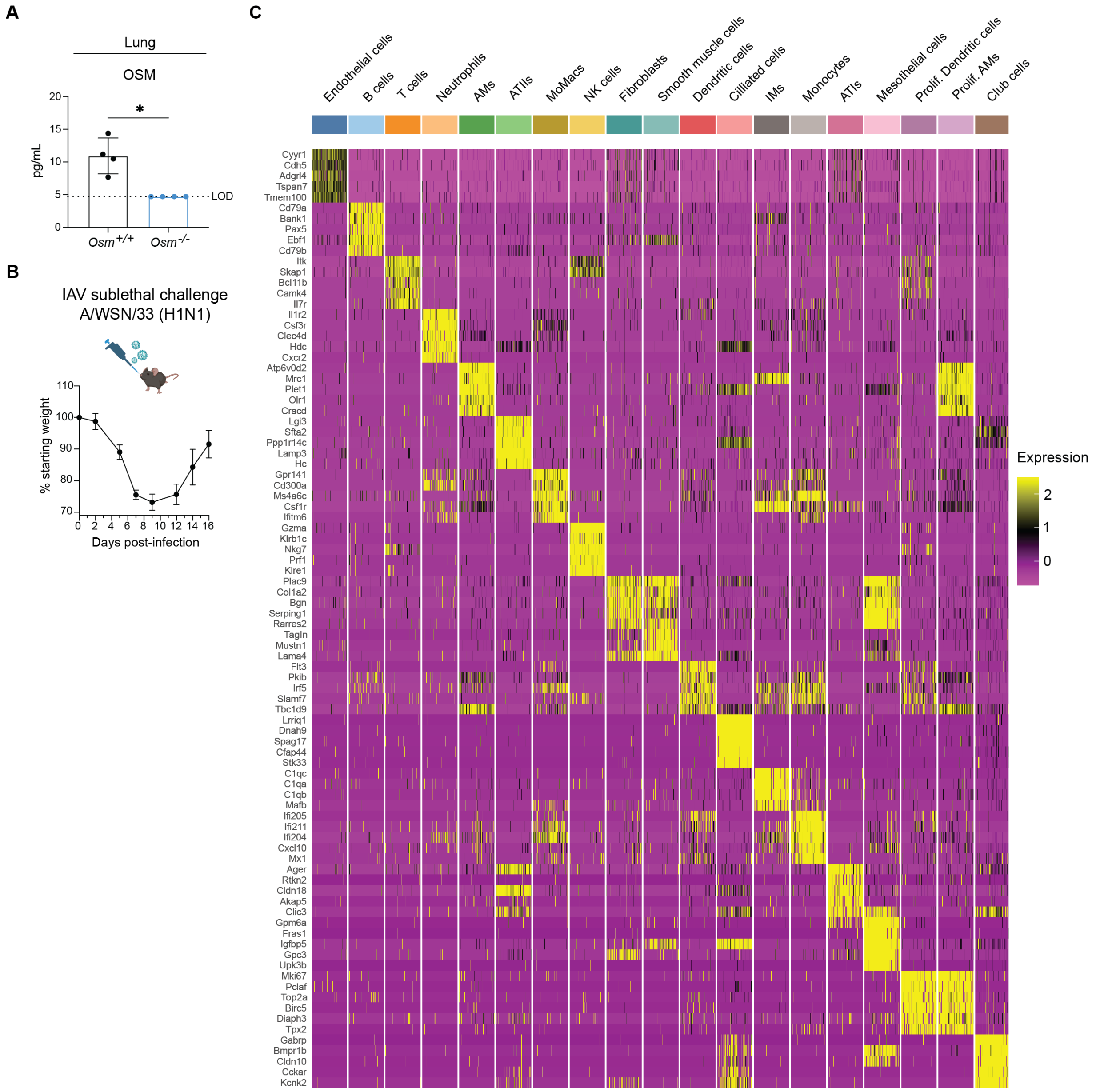

Fig. S1. OSM is present in the lung at steady state, and both immune and nonimmune cell types are identified by scRNA-seq. (**A**) OSM protein levels in homogenized lung tissues were assessed by ELISA (n = 4 mice per group). (**B**) Mice were infected intranasally (i.n.) with 300 plaque-forming units (PFU) of A/WSN/1933 (H1N1) and monitored for body weight daily (n = 4 mice per group). (**C**) Mice were infected i.n. with 225 PFU of A/WSN/1933 (H1N1) or mock-infected with PBS. Lungs were collected from IAV-infected mice at 2 days post-infection (dpi) and mock-infected mice (0 dpi) for single-cell RNA sequencing (scRNA-seq) analysis (n = 2 mice per group). Heat map displaying top-five marker genes (by descending adjusted p-value) of clusters identified in scRNA-seq. Clusters larger than 150 cells were randomly down sampled to 150 cells to populate heatmap. Female mice were used for experiments in this figure. In (A), symbols represent individual mice, bars are mean. In (B), symbols represent mean data. In all

graphs, error bars indicate standard deviation (SD), *$p \leq 0.05$, Mann-Whitney U test for (A). LOD, level of detection.

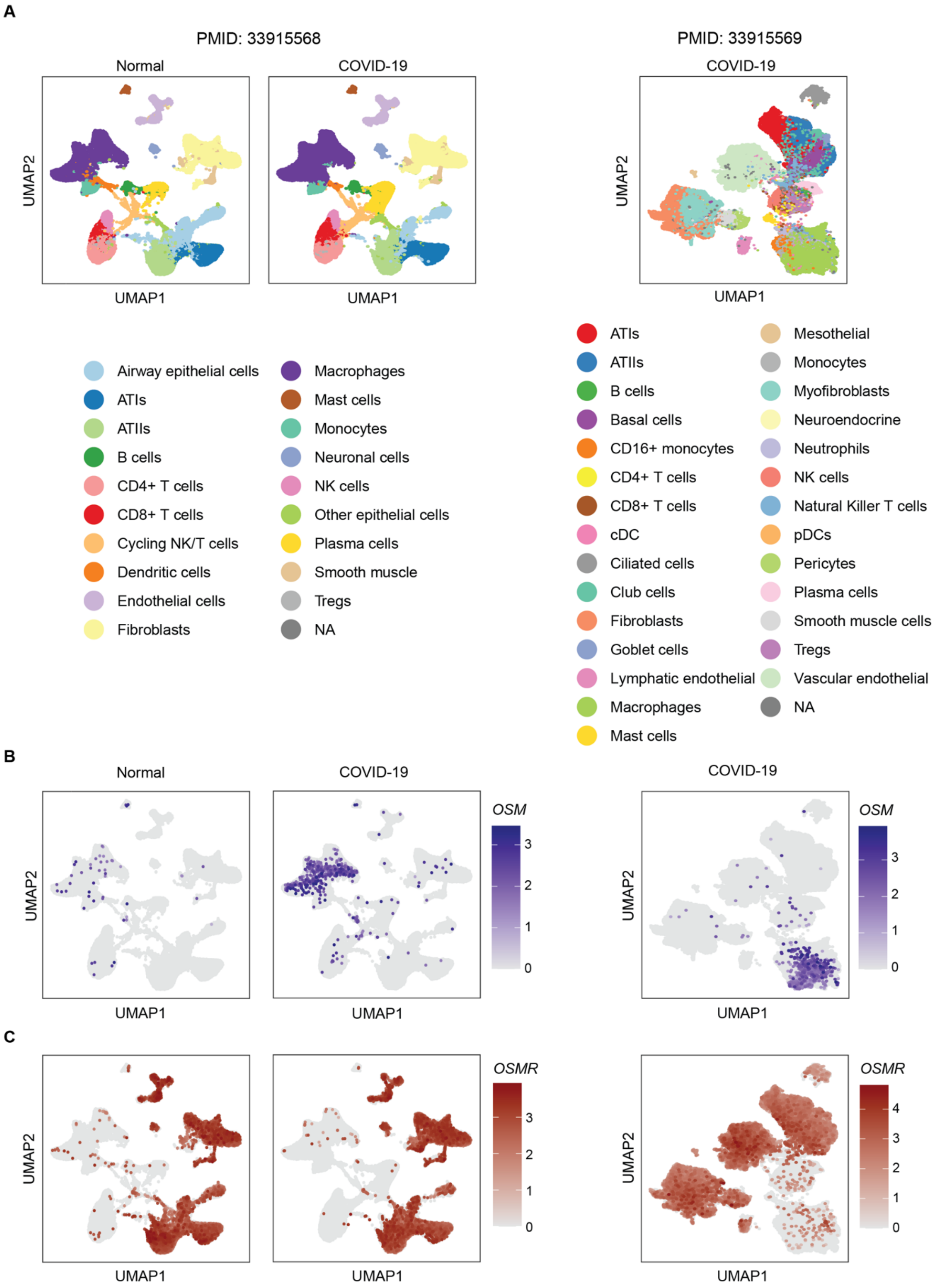

Fig. S2. Human lung *OSM* is expressed in myeloid cells and *OSMR* is expressed in nonimmune cells. (**A**) UMAP clustering and cell cluster annotation of publicly available scRNA-seq data from dissociated lung samples of healthy and COVID-19 patients. Annotations performed as in original manuscripts (*19*, *20*). (**B**) *OSM* expression in annotated cell clusters. **(C)** *OSMR* expression in annotated cell clusters. Scale bar represents log normalized gene expression for (B) and (C).

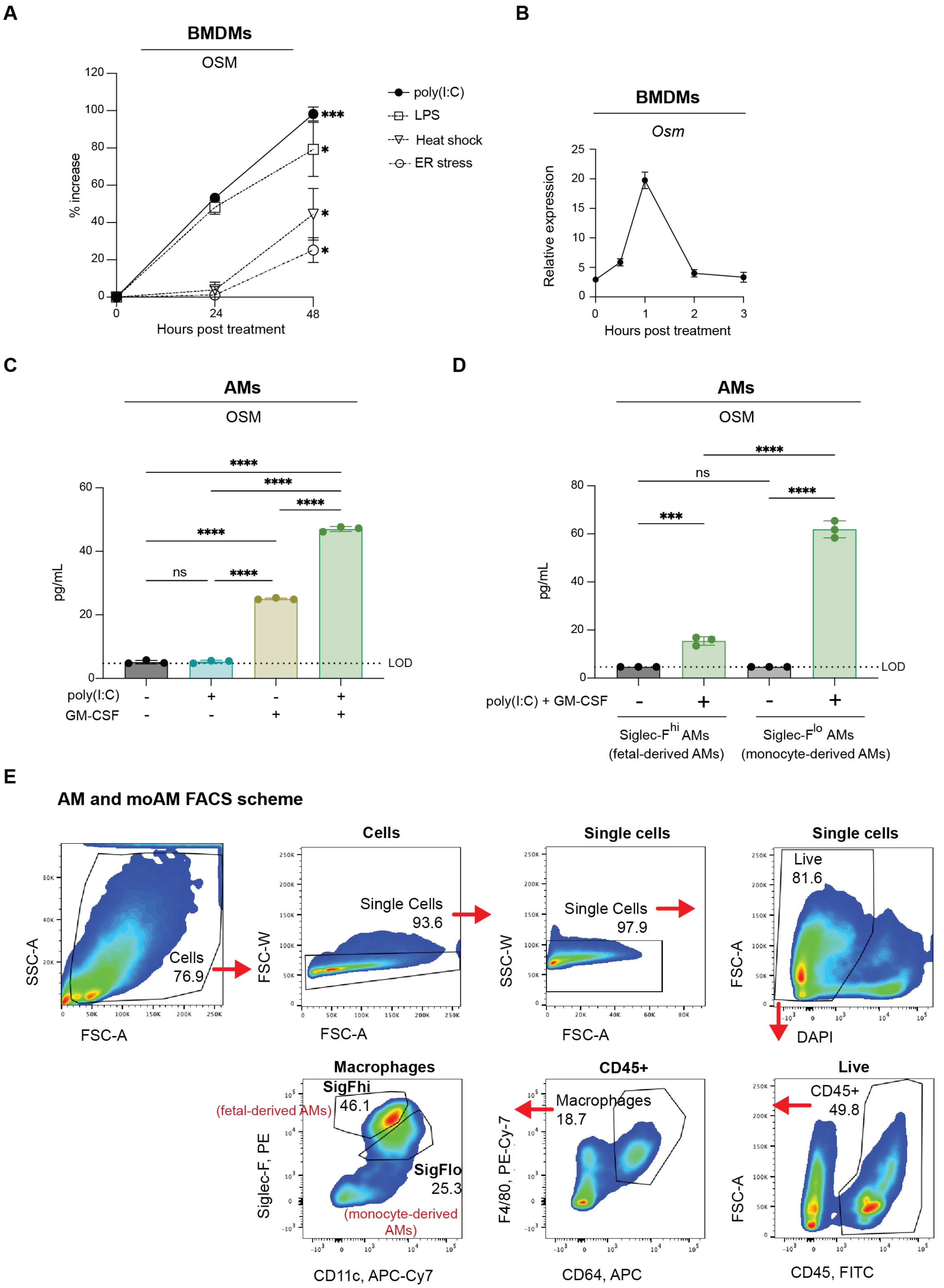

Fig. S3. OSM is induced by myeloid cells in response to diverse signals. (**A**) Bone marrow-derived macrophages (BMDMs) were treated with the indicated PAMPs (50 µg/mL poly(I:C), 10 ng/mL LPS) or stressors (heat shock at 42°C, ER stress induced by 5 µM thapsigargin). Supernatants were

collected at indicated time points, and OSM protein levels were assessed by ELISA (n = 3 samples per group). (**B**) BMDMs were treated with 50 µg/mL poly(I:C) and RNA collected at indicated time points. *Osm* transcript expression was measured via RT-qPCR (n = 3 samples per group). (**C**) AMs were treated with 50 µg/mL poly(I:C), 100 ng/mL GM-CSF, or a combination of both. Supernatants were collected 24 hours after treatment, and OSM protein levels were assessed by ELISA (n = 3 samples per group). (**D**) Female C57BL/6J mice were infected 225 PFU of A/WSN/1933 (H1N1), and lungs collected at 28 dpi. AMs were sorted based on Siglec-F expression (Siglec-F high for fetal-derived AMs and Siglec-F low for monocyte-derived AMs (moAMs) (*18*)). Fetal-derived AMs and moAMs were treated with 50 µg/mL poly(I:C) and 100ng/mL GM-CSF. Supernatants were collected 24 hours after treatment, and OSM protein levels were assessed by ELISA (n = 3 samples per group). (**E**) Representative flow cytometry gating for fetal-derived AMs (CD45+ CD64+ F4/80+ CD11c+ Siglec-F high) and moAMs (CD45+ CD64+ F4/80+ CD11c+ Siglec-F low). Both populations were gated on viable singlets. All data are representative of at least two independent experiments. Relative expression calculated as $(2^{-\Delta Ct})*1000$. In (A) and (B), symbols represent mean data. In (C) and (D), symbols represent individual samples, bars are means. In all graphs, error bars indicate SD, *p≤0.05, ***p≤0.001, ****p≤0.0001, ns = not significant, One sample *t* and Wilcoxon test (48 hours post-treatment versus baseline) for (A), and one-way ANOVA for (C) and (D). LOD, level of detection.

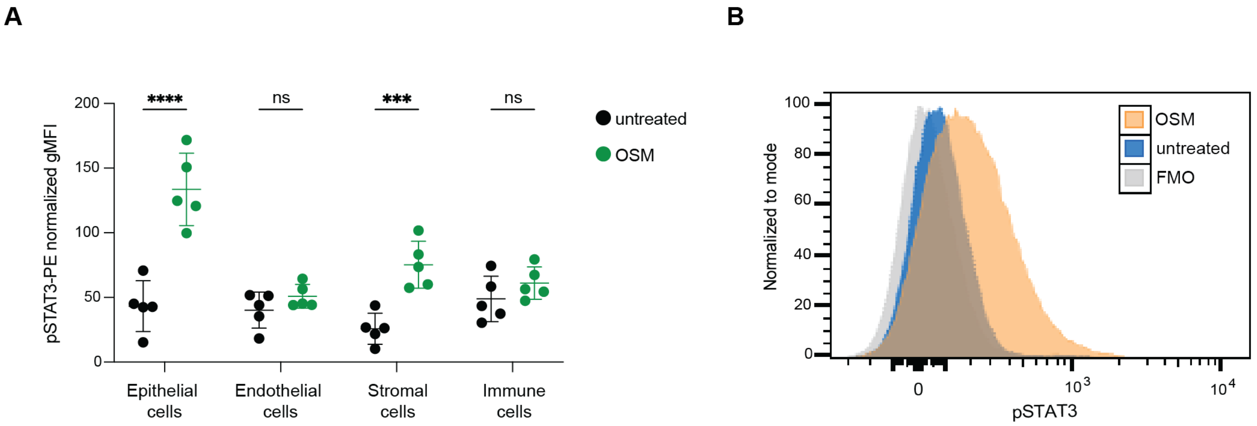

Fig. S4. Lung epithelial cells respond to OSM. Cells isolated from digested lungs of female wild-type mice were *ex vivo* stimulated with rOSM (100 ng/mL) for 30 minutes and stained for surface markers and intracellular pSTAT3 for subsequent flow cytometry analysis. (**A**) Summary representation of pSTAT3 geometric mean fluorescence intensity (gMFI) in indicated populations following stimulation. pSTAT3-PE fluorescence signal was normalized per population by subtracting the PE signal in pSTAT3 fluorescence-minus one controls (FMO) from the stained sample gMFI values. Epithelial cells were defined as EpCAM+ CD45- CD31-, endothelial cells as EpCAM- CD45- CD31+, stromal cells as EpCAM- CD45- CD31-, and immune cells as CD45+, all gated on viable singlets (n = 5 samples per group). (**B**) Representative plot for pSTAT3 staining in epithelial cells following stimulation. All data are representative of at least two independent experiments. In (A) symbols represent individual samples, and error bars indicate SD, ***p≤0.001, ****p≤0.0001, ns = not significant, Two-way ANOVA for (A).

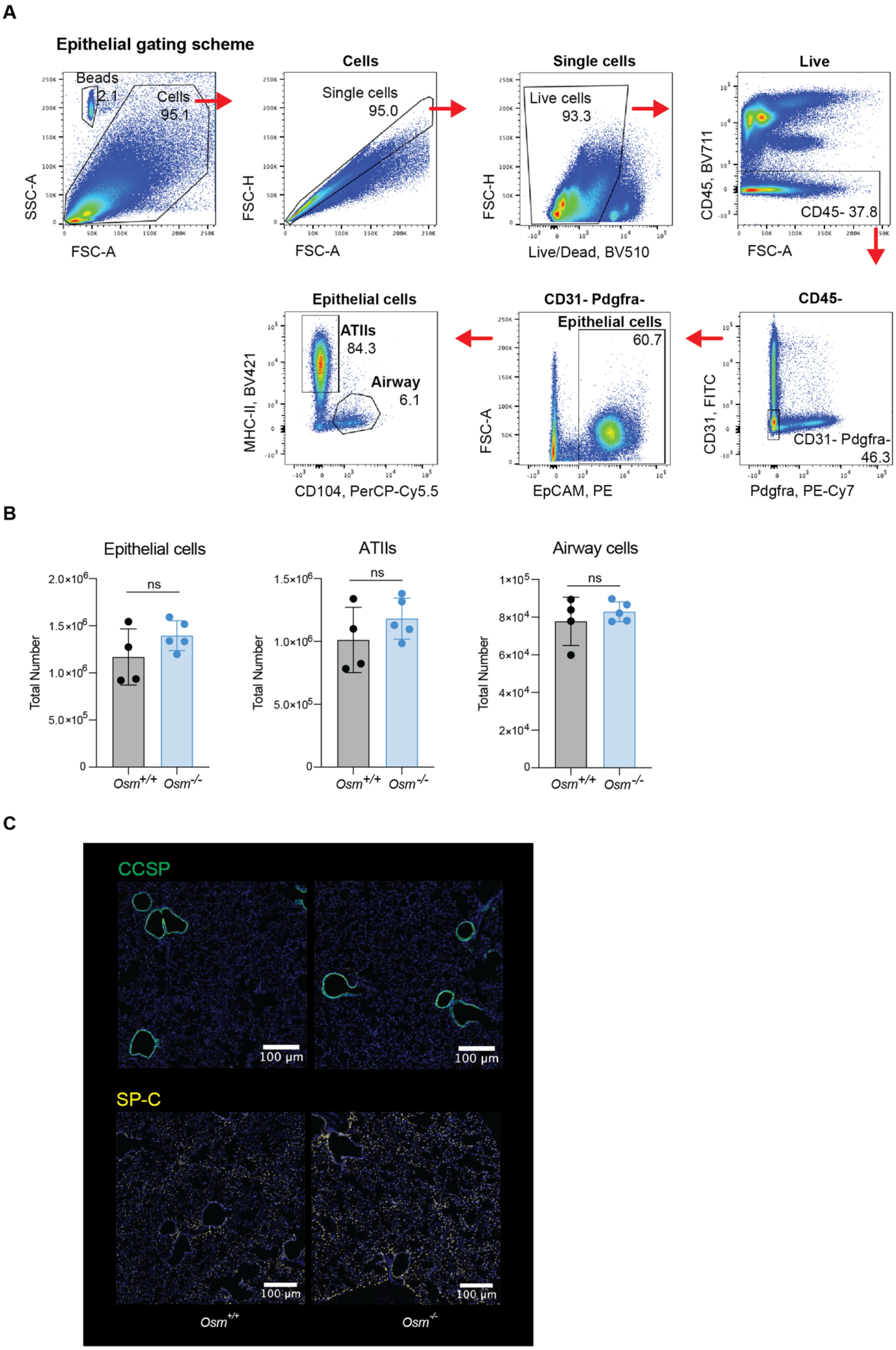
A
Epithelial gating scheme
Beads
2.1
Cells
95.1
SSC-A
FSC-A
Cells
Single cells
95.0
FSC-H
FSC-A
Single cells
Live cells
93.3
FSC-H
Live/Dead, BV510
Live
CD45, BV711
CD45- 37.8
FSC-A
CD45-
CD31, FITC
CD31- Pdgfra-
46.3
Pdgfra, PE-Cy7
CD31- Pdgfra-
Epithelial cells
60.7
FSC-A
EpCAM, PE
Epithelial cells
ATIIs
84.3
Airway
6.1
MHC-II, BV421
CD104, PerCP-Cy5.5
B
Epithelial cells
ATIIs
Airway cells
Total Number
ns
Osm+/+
Osm-/-
C
CCSP
SP-C
100 µm
Osm+/+
Osm-/-

Fig. S5. OSM deficiency does not impact lung epithelial cell numbers at steady state. (**A**) Representative flow cytometry gating strategy for total epithelial cells (CD45-, CD31-, Pdgfra-, EpCAM+), ATIIs (CD45-, CD31-, Pdgfra-, EpCAM+, CD104-, MHCII+), and airway cells (CD45-, CD31-, Pdgfra-, EpCAM+, CD104+, MHCII-). All populations were gated on viable singlets. (**B**) Lungs were processed and total epithelial cell, ATII, and airway cell numbers were determined by flow cytometry (n = 4 – 5 mice per group); representative of at least two independent experiments. (**C**) Representative images of CCSP (green) and SP-C (yellow) staining in mouse lungs (n = 4 – 5 mice per group). A combination of female and male mice was used for experiments in this figure. In (B), symbols represent individual mice, bars are means. Error bars indicate SD, ns = not significant, Unpaired Student's *t* test for (B).

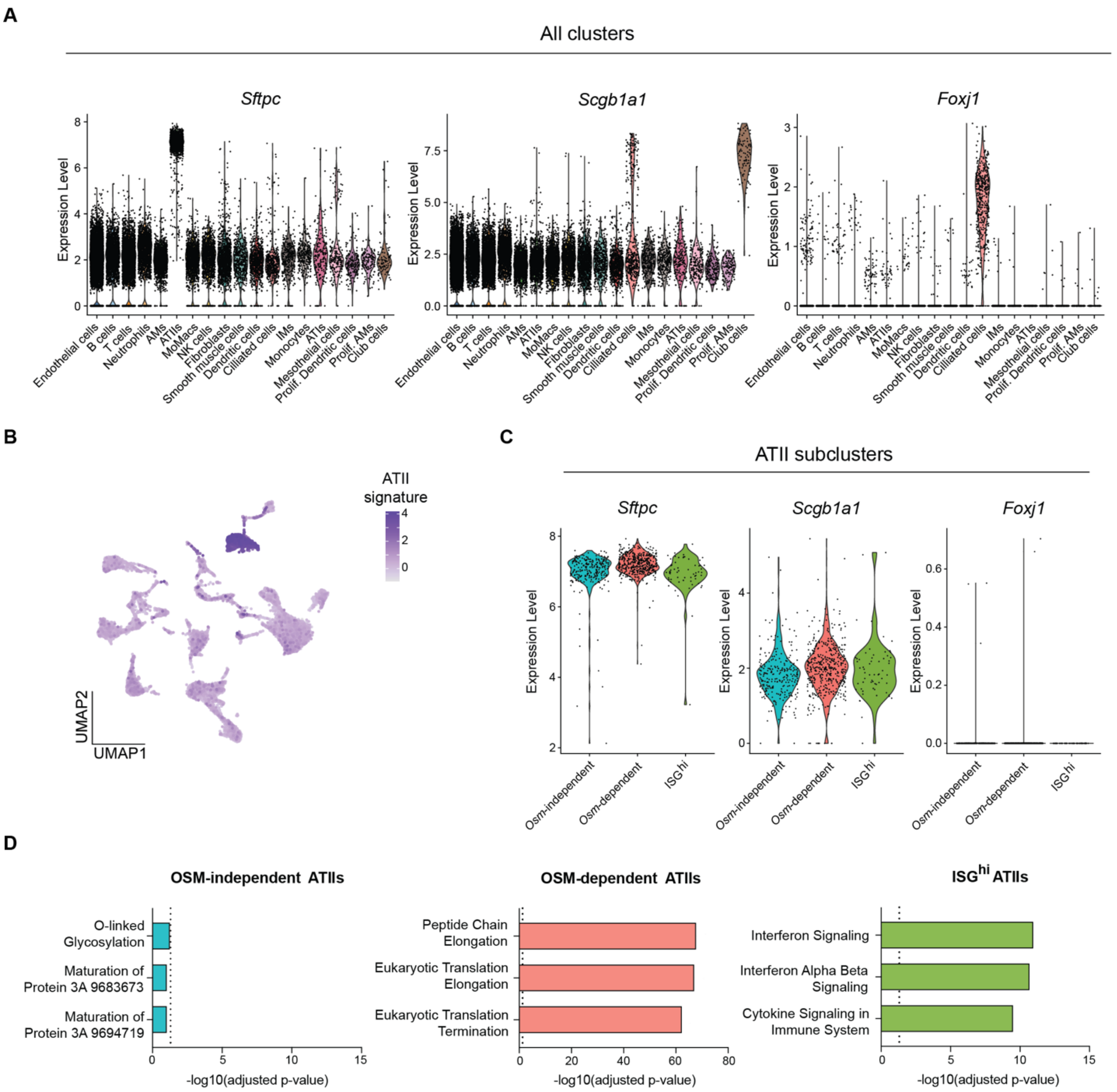

Fig. S6. Gene and pathway enrichment analysis of ATII clusters from scRNA-seq data. *Osm*$^{+/+}$ and *Osm*$^{-/-}$ mice were infected i.n. with 225 pfu of A/WSN/1933 (H1N1) or mock-infected with PBS. Lungs were collected from IAV-infected mice at 2 dpi and from mock-infected mice (0 dpi) for scRNA-seq analysis (n = 2 mice per group). (**A**) Violin plots showing *Sftpc* (left), *Scgb1a1* (middle), and *Foxj1* (right) expression patterns across annotated cell clusters in scRNA-seq data from lungs. (**B**) ATII signature expression across annotated cell clusters (UMAP clustering and cell cluster annotation found in Fig. 1D). Scale bar represents log normalized gene expression. (**C**) Violin plots showing *Sftpc* (left), *Scgb1a1* (middle), and *Foxj1* (right) expression across re-clustered ATII subpopulations. (**D**) Top three pathways identified for each re-clustered ATII subpopulation marker genes at baseline (0 dpi) in wild-type mice, using Reactome pathway enrichment in Enrichr. The dotted line represents the significance threshold for the adjusted p value, set at $p < 0.05$. Female mice were used for experiments in this figure.

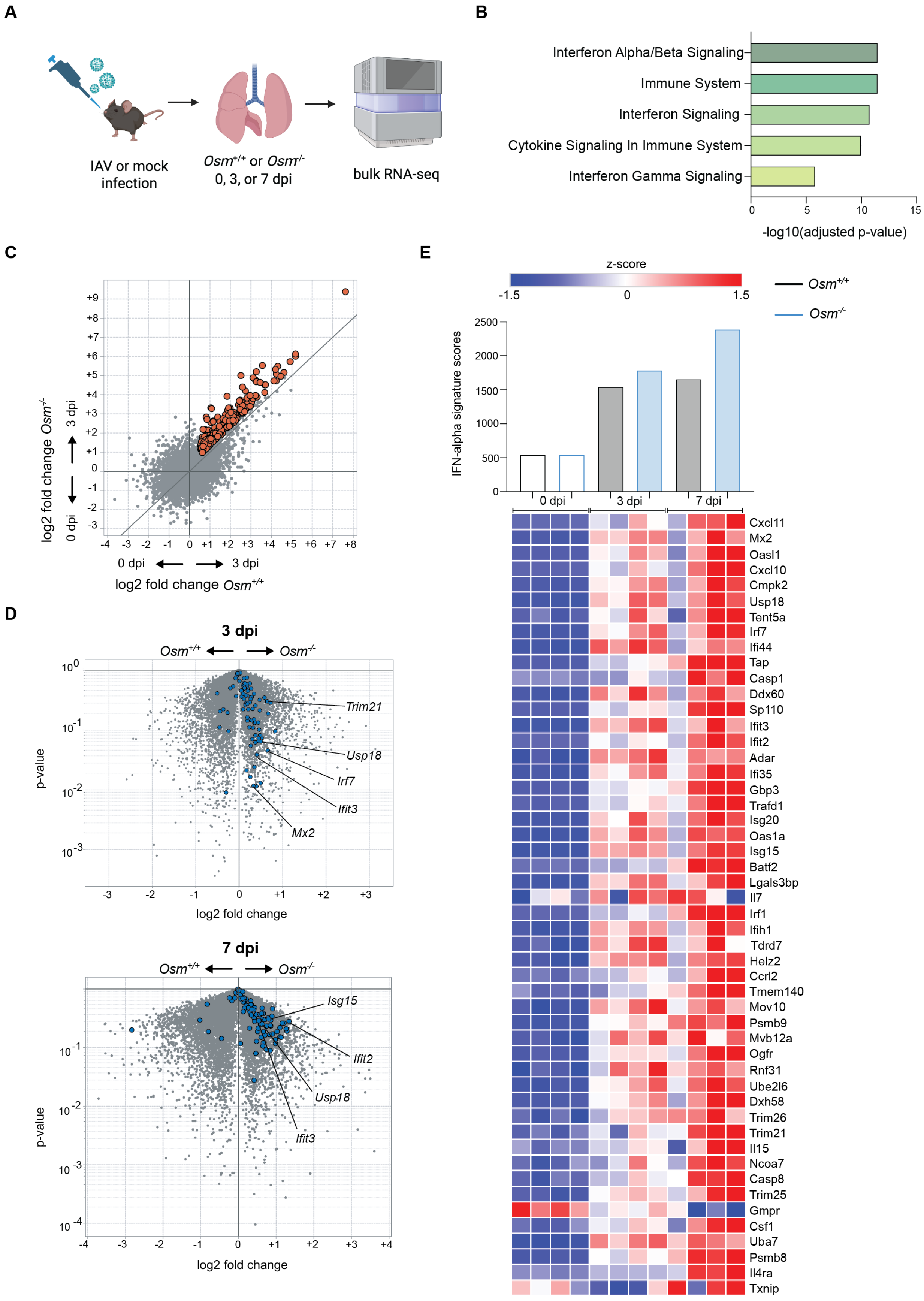
A
IAV or mock infection
Osm+/+ or Osm-/- 0, 3, or 7 dpi
bulk RNA-seq
B
Interferon Alpha/Beta Signaling
Immune System
Interferon Signaling
Cytokine Signaling In Immune System
Interferon Gamma Signaling
-log10(adjusted p-value)
C
log2 fold change Osm-/-
0 dpi
3 dpi
log2 fold change Osm+/+
D
3 dpi
Osm+/+
Osm-/-
Trim21
Usp18
Irf7
Ifit3
Mx2
p-value
log2 fold change
7 dpi
Isg15
Ifit2
Usp18
Ifit3
E
z-score
Osm+/+
Osm-/-
IFN-alpha signature scores
0 dpi
3 dpi
7 dpi
Cxcl11
Mx2
Oasl1
Cxcl10
Cmpk2
Usp18
Tent5a
Irf7
Ifi44
Tap
Casp1
Ddx60
Sp110
Ifit3
Ifit2
Adar
Ifi35
Gbp3
Trafd1
Isg20
Oas1a
Isg15
Batf2
Lgals3bp
Il7
Irf1
Ifih1
Tdrd7
Helz2
Ccrl2
Tmem140
Mov10
Psmb9
Mvb12a
Ogfr
Rnf31
Ube2l6
Dxh58
Trim26
Trim21
Il15
Ncoa7
Casp8
Trim25
Gmpr
Csf1
Uba7
Psmb8
Il4ra
Txnip

Fig. S7. OSM-deficient mice exhibit an enhanced IFN response during IAV infection. Mice were infected i.n. with 225 PFU of A/WSN/1933(H1N1) or mock-infected with PBS. Lungs were collected from IAV-infected mice at 3 or 7 dpi and mock-infected mice (0 dpi) for bulk RNA-seq analysis (n = 2 mice per group). (**A**) Schematic representation of experimental design. (**B**) Transcriptional analysis of whole lung gene expression using Reactome pathway enrichment in Enrichr on genes that were at least 0.5 log2 fold change upregulated in both *Osm*$^{+/+}$ and *Osm*$^{-/-}$ mice at 3 dpi versus 0 dpi and 0.25 log2 fold change more differentially upregulated in IAV-infected *Osm*$^{-/-}$ mice as compared to *Osm*$^{+/+}$ mice. (**C**) FC/FC plots comparing gene expression values at 3 dpi versus 0 dpi, in *Osm*$^{-/-}$ versus *Osm*$^{+/+}$ mice. Highlighted genes were induced at least 1.5x fold in 3 dpi/0 dpi lungs for both *Osm*$^{+/+}$ and *Osm*$^{-/-}$ mice and had 1.25x higher fold change values in *Osm*$^{-/-}$ compared to *Osm*$^{+/+}$ mice. (**D**) Volcano plots illustrating expression of MSigDB hallmark interferon alpha response signature genes between *Osm*$^{+/+}$ and *Osm*$^{-/-}$ mice at 3 dpi (top) and 7 dpi (bottom). (**E**) Expression of MSigDB hallmark IFN-α response signature genes in whole lung. Average expression of IFN-α response genes (top) and heatmap of selected transcripts from the MSigDB hallmark IFN-α response signature gene set (bottom). The top 50 genes that displayed enhanced upregulation in *Osm*$^{-/-}$ mice at 3 dpi, as identified in (C), were selected for display. Color scale reflects row z-score values. Female mice were used for experiments in this figure.

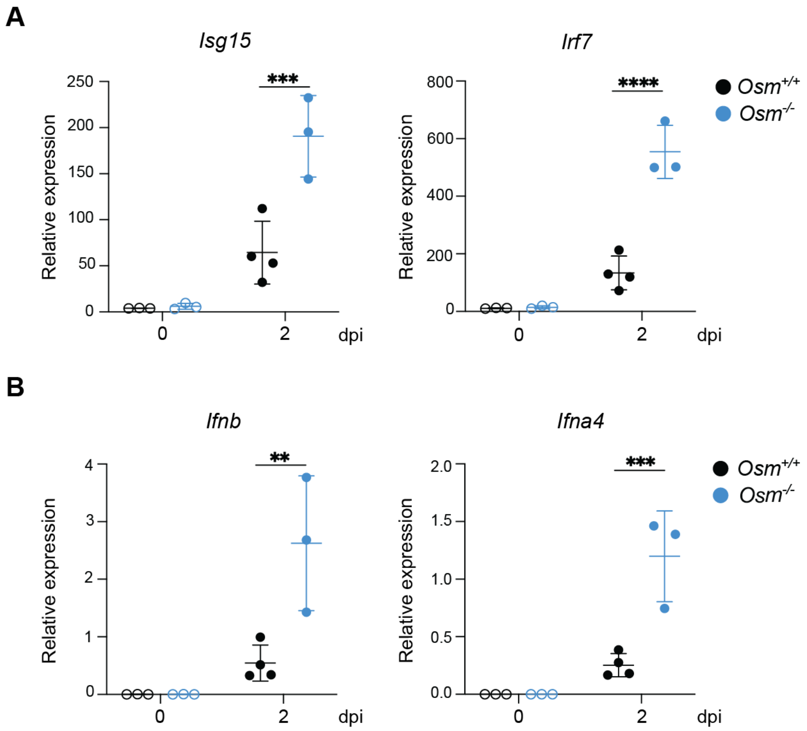

Fig. S8. OSM deficiency results in elevated IFN transcript levels during IAV infection. Mice were infected i.n. with 225 PFU of A/WSN/1933(H1N1) or mock-infected with PBS. Whole lung RNA was collected from IAV-infected mice at 2 dpi and mock-infected mice (0 dpi). (**A**) *Isg15* and *Irf7* transcript expression was measured via RT-qPCR (n = 3 – 4 mice per group). (**B)** *Ifnb* and *Ifna4* transcript expression was measured via RT-qPCR (n = 3 – 4 mice per group). All data are representative of at least two independent experiments. Female mice were used for experiments in this figure. Relative expression calculated as ($2^{-\Delta Ct}$)*1000. Symbols represent individual mice, and error bars indicate SD, **p≤0.01, ***p≤0.001, ****p≤0.0001, Two-way ANOVA.

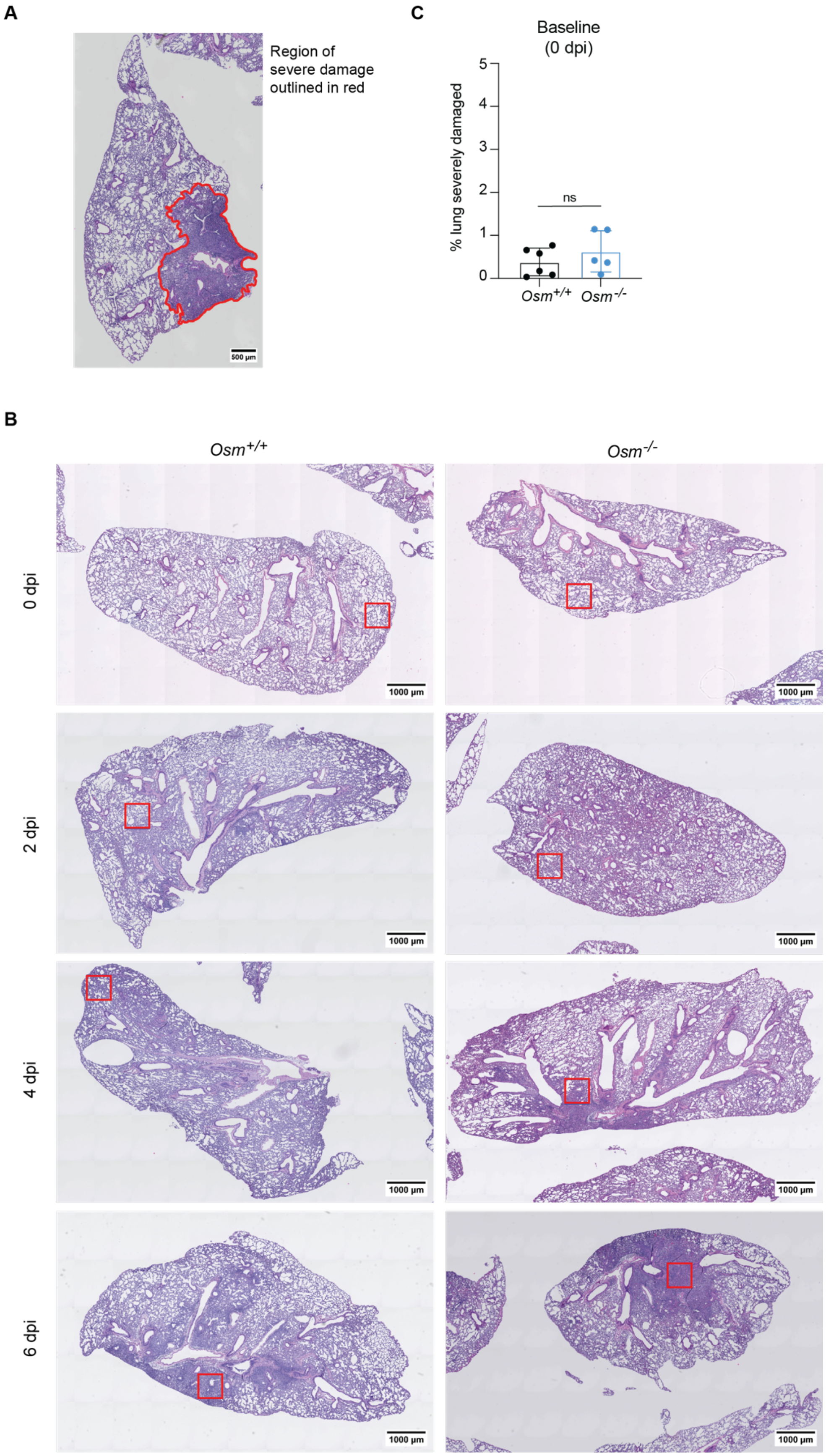
A
Region of
severe damage
outlined in red
500 µm
C
Baseline
(0 dpi)
% lung severely damaged
5
4
3
2
1
0
ns
Osm+/+
Osm-/-
B
Osm+/+
Osm-/-
0 dpi
2 dpi
4 dpi
6 dpi
1000 µm

Fig. S9. OSM deficiency results in increased immunopathology during IAV infection. (**A – B**) Mice were infected i.n. with 225 pfu of A/WSN/1933 (H1N1). Representative image showing an example of a region defined as severe lung damage, used to train QuPath for damage classification (A). Representative histological images of mouse lungs at baseline and at various time points during IAV infection; red squares indicate regions magnified in Fig. 2D (B). (**C**) Histological scoring of baseline (uninfected) lungs from $Osm^{+/+}$ and $Osm^{-/-}$ mice was performed to quantify severe damage. Lung sections from $Osm^{+/+}$ mice at steady state were used to define healthy regions and to train QuPath for classification (n = 5 – 6 mice per group, 1 – 3 fields per mouse). Female mice were used for experiments in this figure. Symbols represent individual mice, bars are means. Error bars indicate SD, ns = not significant, Unpaired Student's *t* test for (C).

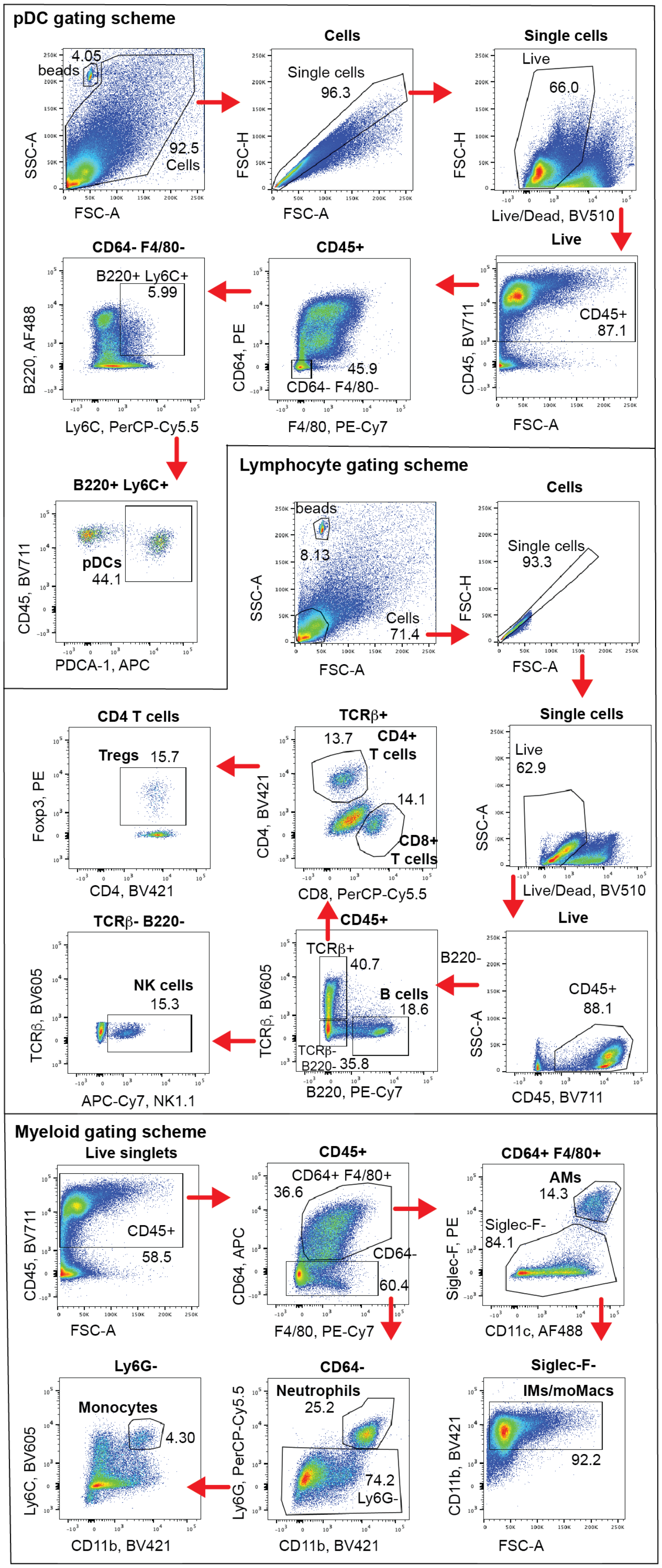

pDC gating scheme
beads
4.05
92.5
Cells
SSC-A
FSC-A
Cells
Single cells
96.3
FSC-H
FSC-A
Single cells
Live
66.0
FSC-H
Live/Dead, BV510
Live
CD45+
87.1
CD45, BV711
FSC-A
CD45+
45.9
CD64- F4/80-
CD64, PE
F4/80, PE-Cy7
CD64- F4/80-
B220+ Ly6C+
5.99
B220, AF488
Ly6C, PerCP-Cy5.5
B220+ Ly6C+
pDCs
44.1
CD45, BV711
PDCA-1, APC
Lymphocyte gating scheme
beads
8.13
Cells
71.4
SSC-A
FSC-A
Cells
Single cells
93.3
FSC-H
FSC-A
Single cells
Live
62.9
SSC-A
Live/Dead, BV510
Live
CD45+
88.1
SSC-A
CD45, BV711
CD45+
TCRβ+
40.7
B cells
18.6
TCRβ-
B220-
35.8
B220-
TCRβ, BV605
B220, PE-Cy7
TCRβ+
13.7
CD4+
T cells
14.1
CD8+
T cells
CD4, BV421
CD8, PerCP-Cy5.5
CD4 T cells
Tregs
15.7
Foxp3, PE
CD4, BV421
TCRβ- B220-
NK cells
15.3
TCRβ, BV605
APC-Cy7, NK1.1
Myeloid gating scheme
Live singlets
CD45+
58.5
CD45, BV711
FSC-A
CD45+
CD64+ F4/80+
36.6
CD64-
60.4
CD64, APC
F4/80, PE-Cy7
CD64+ F4/80+
AMs
14.3
Siglec-F-
84.1
Siglec-F, PE
CD11c, AF488
Siglec-F-
IMs/moMacs
92.2
CD11b, BV421
FSC-A
CD64-
Neutrophils
25.2
74.2
Ly6G-
Ly6G, PerCP-Cy5.5
CD11b, BV421
Ly6G-
Monocytes
4.30
Ly6C, BV605
CD11b, BV421

Fig. S10. Gating strategies for flow cytometry analysis of lung immune infiltrates during IAV infection. Representative flow cytometry gating strategy for lung immune infiltrates. Populations were gated as follows: pDCs (CD45+ CD64- F4/80- B220+ Ly6C+ PDCA1+), CD4 T cells (CD45+ TCRB+ B220- CD4+ CD8-), CD8 T cells (CD45+ TCRB+ B220- CD4- CD8+), Tregs (CD45+ TCRB+ B220- CD4+ CD8- FOXP3+), NK cells (CD45+ B220- TCRB- NK1.1+), B cells (CD45+ TCRB- B220+), AMs (CD45+ CD64+ F4/80+ Siglec-F+ CD11c+), IMs/moMacs (CD45+ CD64+ F4/80+ Siglec-F- CD11b+), neutrophils (CD45+ CD64- Ly6G+ CD11b+), monocytes (CD45+ CD64- Ly6G- CD11b+ Ly6C+). All populations were gated on viable singlets.

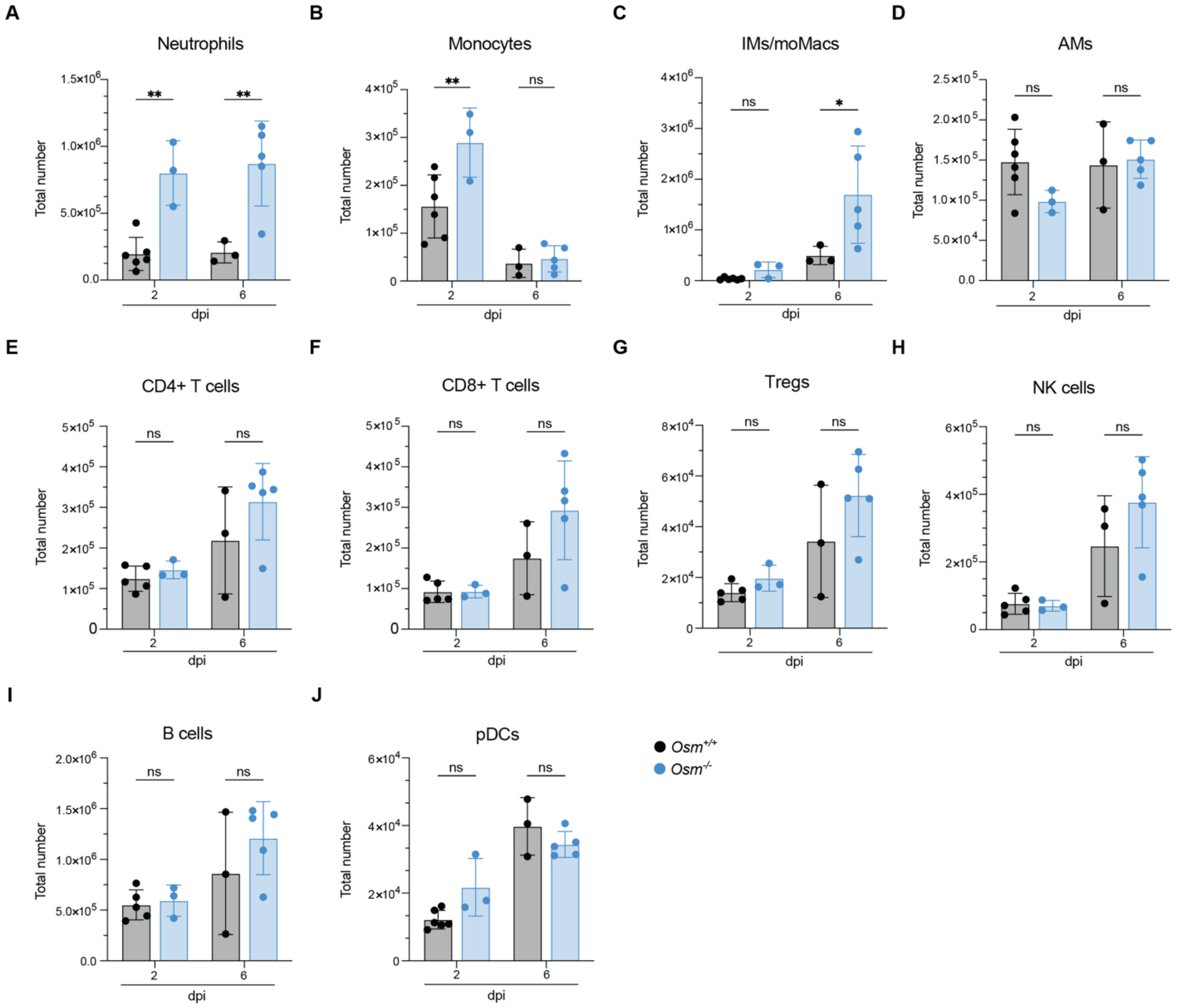

Fig. S11. Flow cytometry analysis of lung immune infiltrates during IAV infection. Mice were infected i.n. with 450 PFU of A/WSN/1933 (H1N1). Lung immune infiltrates were quantified by flow cytometry at 2 and 6 dpi (n = 3 – 6 mice per group). Representative flow cytometry gating strategies for all populations found in fig. S10. (**A**) Neutrophils, (**B**) Monocytes, (**C**) IMs/moMacs, (**D**) AMs, (**E**) CD4+ T cells, (**F**) CD8+ T cells, (**G**) Tregs, (**H**) NK cells, (**I**) B cells and (**J**) pDCs. Male mice were used for experiments in this figure. Symbols represent individual mice, bars are means. Error bars indicate SD, *p≤0.05, **p≤0.01, ns = not significant, Two-way ANOVA.

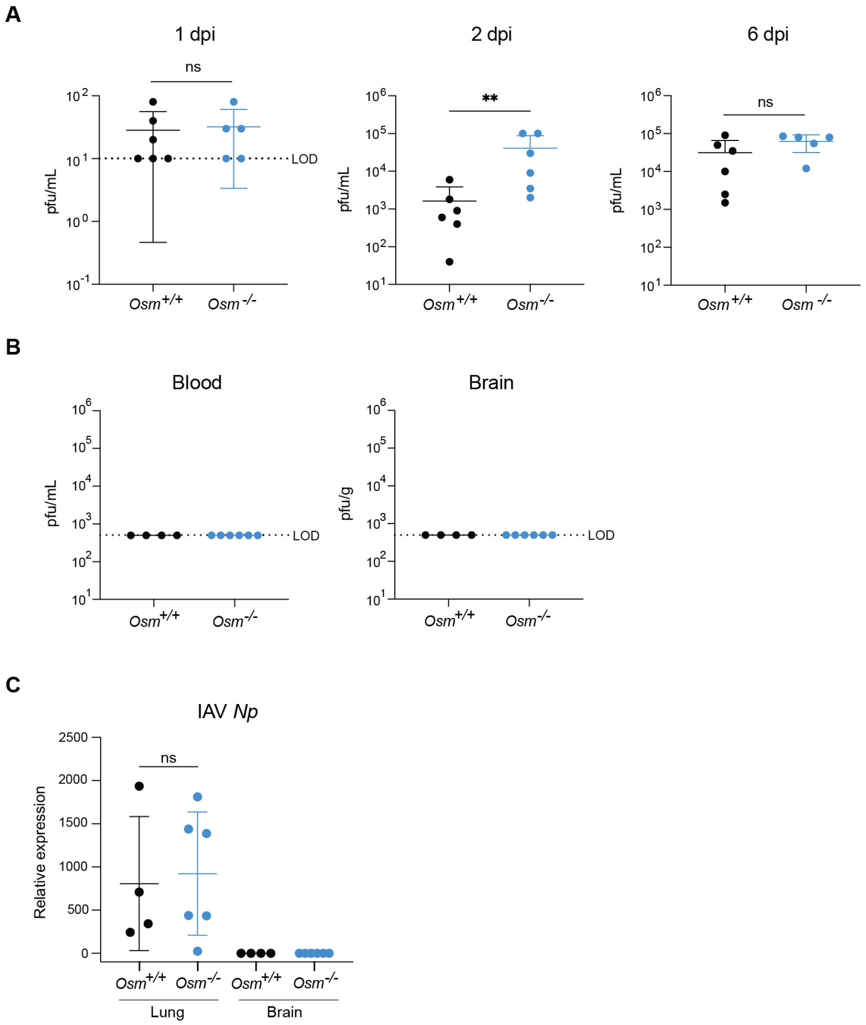

Fig. S12. IAV is not disseminated in OSM-deficient mice. Mice were infected i.n. with 225 PFU of A/WSN/1933 (H1N1). (**A**) BALF was harvested and PFU determined at indicated time points (n = 5 – 6 mice per group). (**B**) Blood and brain were harvested, homogenized, and PFU determined at 6 dpi (n = 4 – 6 mice per group). (**C**) RT-qPCR analysis of IAV transcripts encoding nucleoprotein (*Np*) in whole lung or brain homogenate at 6 dpi (n = 4 – 6 mice per group); representative of at least two independent experiments. Female mice were used for experiments in this figure. Relative expression calculated as $(2^{-\Delta Ct})$*1000. Symbols represent individual mice, and error bars indicate SD, **p≤0.01, ns = not significant, Unpaired Student's *t* test or Mann-Whitney U test for (A), unpaired Student's *t* test for (C). LOD, level of detection.

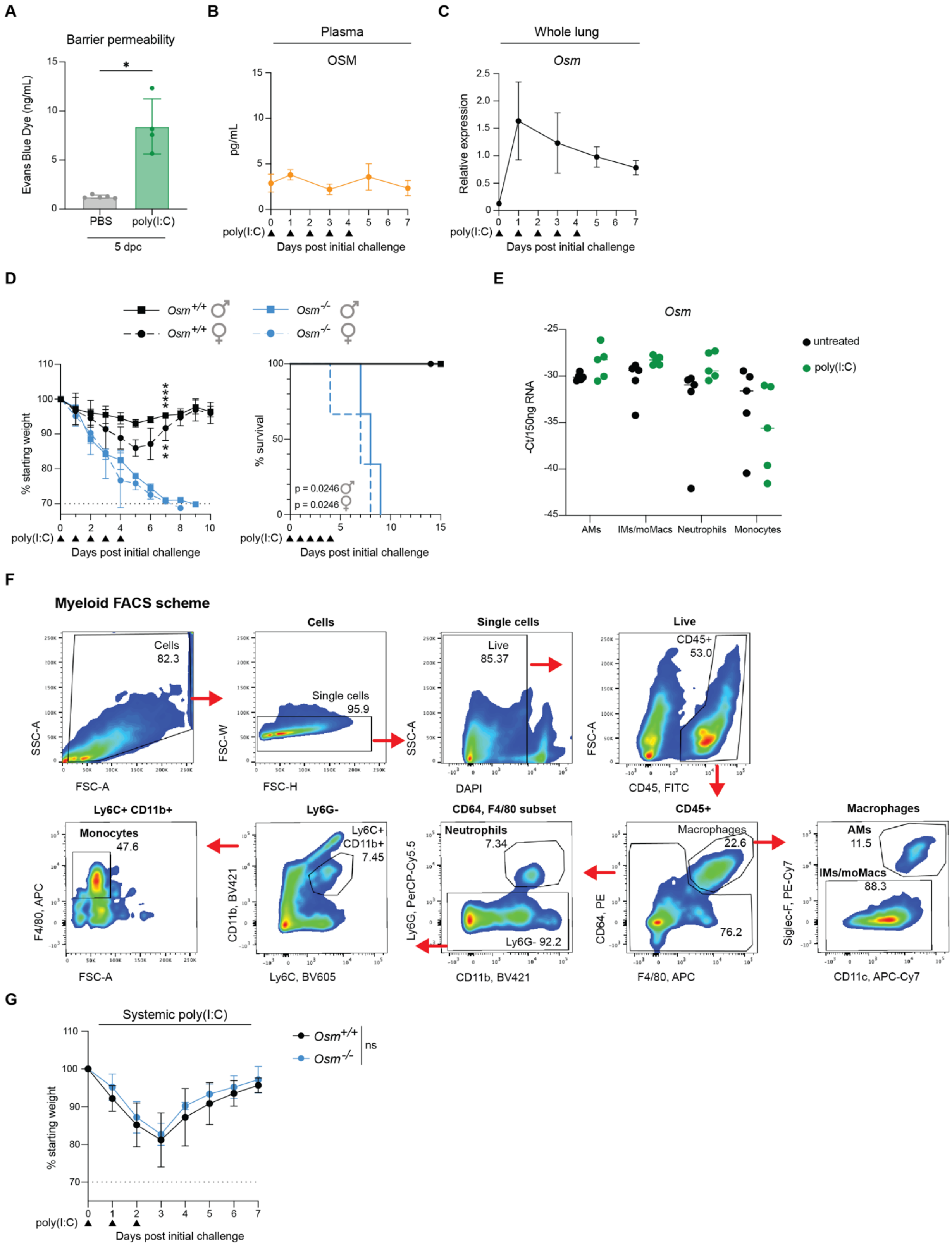

Fig. S13. OSM is required for survival following local poly(I:C) challenge. (A) Mice were treated intratracheally (i.t.) with poly(I:C) or PBS daily for five consecutive days. Lung barrier

permeability was assessed using an Evans Blue Dye assay on BALF at 5 days post initial challenge (dpc) (n = 4 – 5 mice per group). (**B – D**) Mice were treated i.t. with poly(I:C) daily for five consecutive days. Plasma was collected for analysis of OSM protein levels via ELISA at the indicated time points (B) (n = 4 – 5 mice per group); representative of two independent experiments. Whole lung RNA was collected at indicated time points, and *Osm* transcript expression measured via RT-qPCR (C) (n = 4 – 5 mice per group). Mice were monitored daily for percent initial body weight (left panel) and survival (right panel) (D) (n = 3 mice per group); representative of at least two independent experiments. (**E**) Mice were treated i.t. with poly(I:C) for five consecutive days. Myeloid populations from wild-type mice collected at 7 dpc were sorted and *Osm* transcript expression was measured via RT-qPCR (n = 5 mice per group). (**F**) Representative flow cytometry gating strategy for sorted myeloid populations. Populations were gated as follows: neutrophils (CD45+ CD64- Ly6G+ CD11b+), monocytes (CD45+ CD64- Ly6G- CD11b+ Ly6C+ F4/80+), IMs/moMacs (CD45+ CD64+ F4/80+ Siglec-F-), and AMs (CD45+ CD64+ F4/80+ CD11c+ Siglec-F+). All populations were gated on viable singlets. (**G**) Mice were treated intraperitoneally (i.p.) with 100 µg of poly(I:C) daily for three consecutive days and body weight was monitored (n = 6 mice per group). Female mice were used for (A), (B), (C), and (E), and male mice for (G). Relative expression calculated as $(2^{-\Delta Ct})*1000$. -Ct values normalized by RNA input. In (A), symbols represent individual mice, bars are means. In (B - D) and (G), symbols represent mean data. In (E), symbols represent individual samples. In all graphs, error bars indicate SD, *p≤0.05, **p≤0.01, ****p≤0.0001, ns = not significant, Mann-Whitney U test for (A), two-way ANOVA up to day 7 for (D, left panel) and (G), log-rank Mantel-Cox test for (D, right panel).

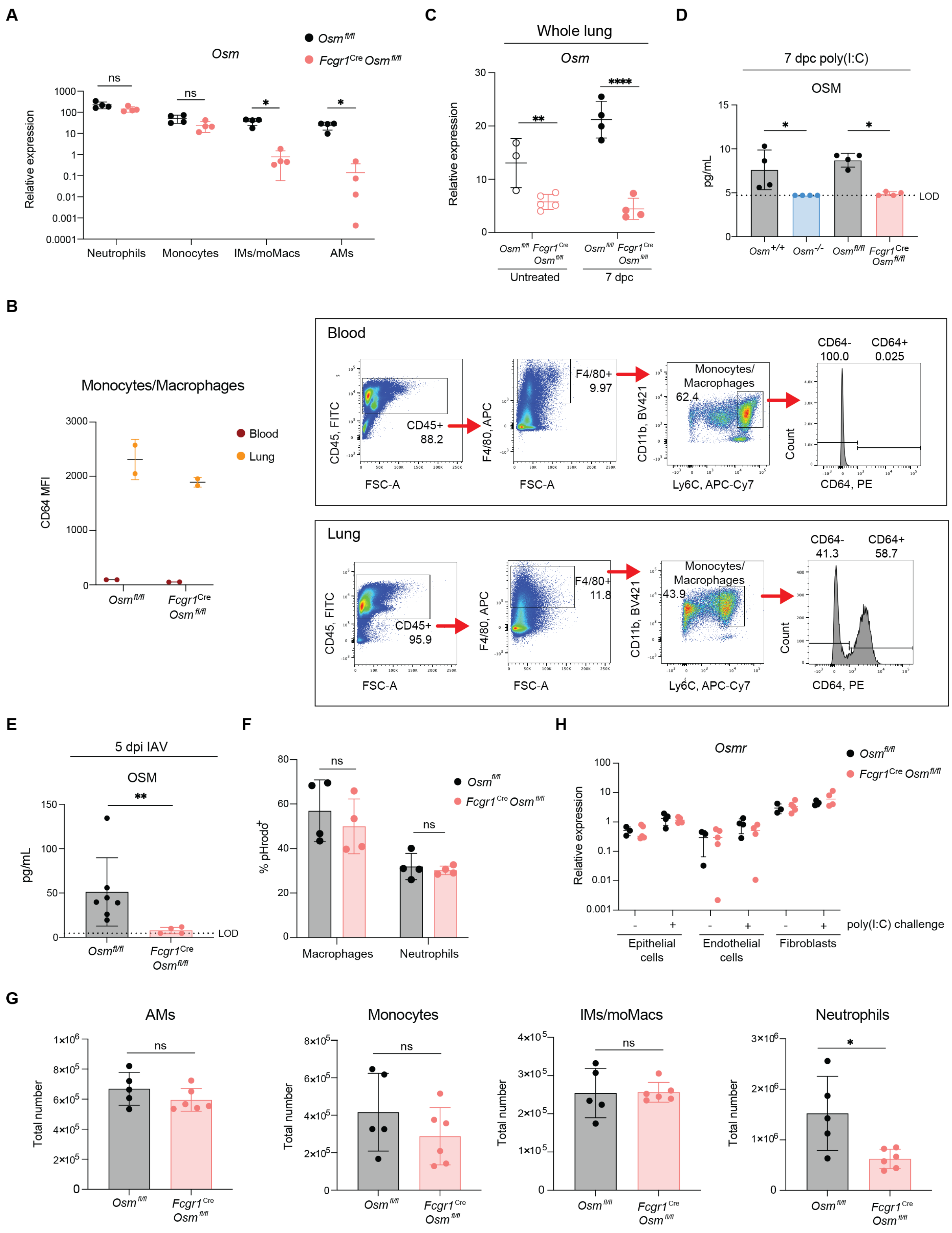

Fig. S14. Validation of macrophage-specific OSM-deficient mouse model. (A) Mice were treated i.t. with poly(I:C) daily for five consecutive days. Myeloid cells were sorted from lungs of mice at 7 dpc and *Osm* transcript expression measured via RT-qPCR (n = 4 mice per group).

Representative flow cytometry gating strategy for sorted myeloid populations found in fig. S13F. (**B**) Blood and lungs of mice at steady state were processed for flow cytometry analysis of monocytes/macrophages (CD45+ F4/80+ CD11b+ Ly6C+ viable singlets). CD64 MFI quantification (left panel) (n = 2 mice per group), and representative flow cytometry gating strategy and CD64 MFI expression (right panel). (**C**) Mice were treated i.t. with poly(I:C) daily for five consecutive days or left untreated. Whole lung *Osm* transcripts were quantified via RT-qPCR at baseline and at 7 dpc (n = 3 – 5 mice per group). (**D**) Mice were treated i.t. with poly(I:C) daily for five consecutive days. BALF was collected at 7 dpc and OSM protein levels were detected by ELISA (n = 4 mice per group). (**E**) Mice were infected i.n. with 225 PFU of A/WSN/1933 (H1N1). BALF was collected at 5 dpi and OSM protein levels were detected by ELISA (n = 4 – 7 mice per group). (**F**) Mice were treated i.t. with 50 µg of pHrodo *E. coli* beads and lungs were processed for flow cytometry analysis; populations were gated as follows: neutrophils (CD45+ CD64- F4/80- Ly6G+ CD11b+), and macrophages (CD45+ CD64+ F4/80+). All populations were gated on viable singlets (n = 4 mice per group). (**G**) Baseline lungs were processed and myeloid cell numbers determined by flow cytometry (n = 5 – 6 mice per group). Representative flow cytometry gating strategies for myeloid cells found in fig. S10. (**H**) Mice were treated i.t. with poly(I:C) or PBS daily for five consecutive days. Epithelial cells (CD45- Pdgfra- CD31- EpCAM+), endothelial cells (CD45- Pdgfra- CD31+), and fibroblasts (CD45- CD31- Pdgfra+) were sorted at 7 dpc and RT-qPCR was performed to quantify *Osmr* transcript expression levels (n = 3 -5 mice per group). A combination of female and male mice was used for experiments in this figure. Relative expression calculated as $(2^{-\Delta Ct})*1000$. In (A - C) and (H), symbols represent individual mice. In (D - G), symbols represent individual mice, bars are mean. In all graphs, error bars indicate SD, *p≤0.05, **p≤0.01, ****p≤0.0001, ns = not significant. Mann-Whitney U test for (A) and (E), Two-way ANOVA for (C), non-parametric one-way ANOVA for (D), unpaired Student's *t* test for (F), Mann-Whitney U test or unpaired Student's *t* test for (G). LOD, level of detection.

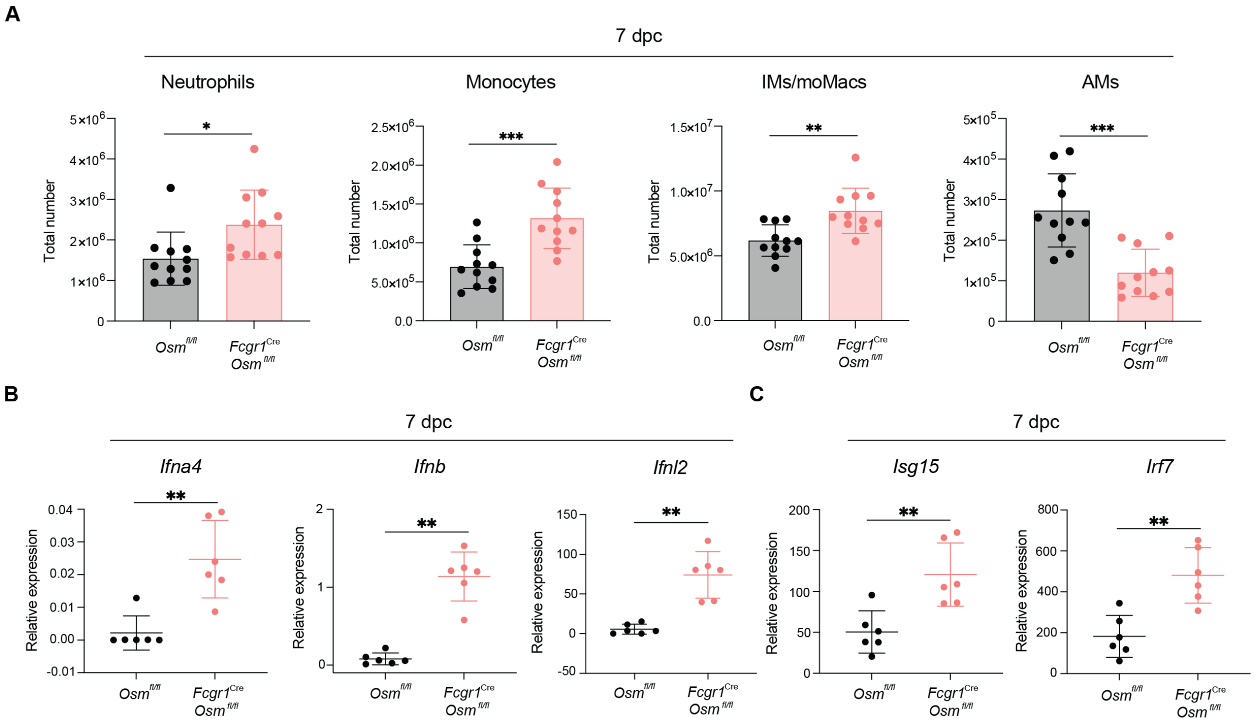

Fig. S15. Macrophage-specific deletion of *Osm* results in elevated IFN transcript levels and immune infiltration following poly(I:C) challenge. Mice were treated i.t. with poly(I:C) daily for 5 consecutive days. (**A**) Lungs were processed at 7 dpc, and myeloid cell numbers determined by flow cytometry (n = 11 mice per group); pooled from three independent experiments. Representative flow cytometry gating strategies for myeloid cells found in fig. S10. (**B - C**) Whole lung RNA was collected and RT-qPCR performed for *Ifna4*, *Ifnb*, and *Ifnl2* (B) and for *Isg15* and Irf7 (C) (n = 6 mice per group). A combination of male and female mice was used for experiments in this figure. Relative expression calculated as $(2^{-\Delta Ct})*1000$. In (A), symbols represent individual mice, bars are means. In (B) and (C), symbols represent individual mice. In all graphs, error bars indicate SD, *p≤0.05, **p≤0.01, ***p≤0.001, Mann-Whitney U test for (B), unpaired Student's *t* test for (A) and (C).

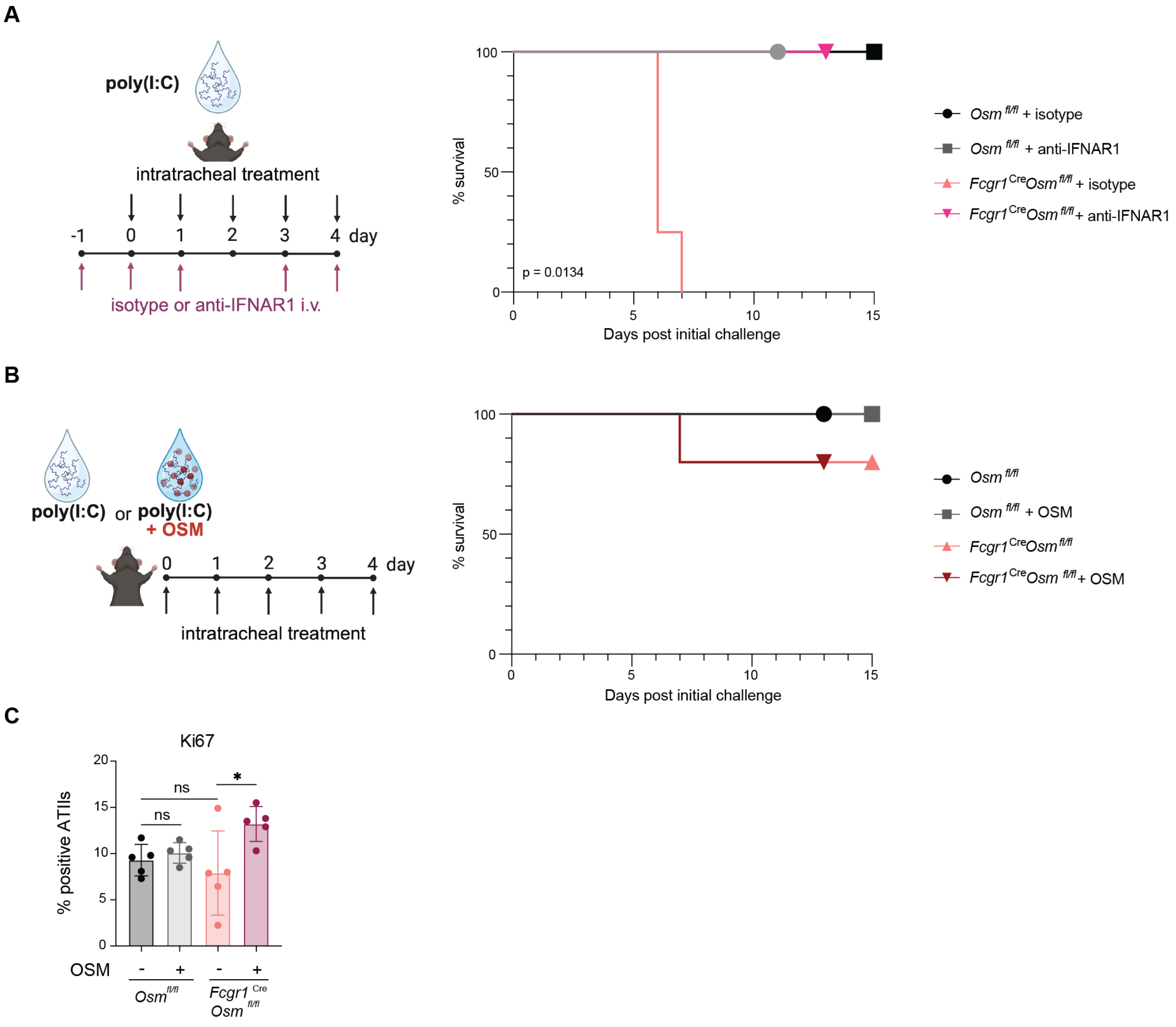

Fig. S16. Blockade of IFN-I signaling rescues survival of macrophage-specific OSM-deficient mice during poly(I:C) challenge. (**A**) Mice were treated i.t. with poly(I:C) daily for 5 consecutive days. In addition, mice were treated intravenously (i.v.) with either 200 µg of anti-mouse IFNAR1 or isotype control antibody diluted in 100 µl PBS on day -1, 0, 1, 3 and 4 after the first poly(I:C) challenge (n = 3 – 4 mice per group); representative of at least two independent experiments. Mice were monitored for survival. (**B - C**) Mice were treated i.t. with 1 µg of mouse rOSM in addition to poly(I:C) daily for five consecutive days. Mice were monitored for survival (B) (n = 4 – 5 mice per group); representative of at least two independent experiments. Lungs were processed at 7 dpc, and Ki67+ ATII proportions determined by flow cytometry (C) (n = 5 mice per group). Representative flow cytometry gating strategy for ATIIs found in fig. S5A. Female mice were used for experiments in this figure. In (C), symbols represent individual mice, bars are means. Error bars indicate SD, *$p \leq 0.05$, ns = not significant. Log-rank Mantel Cox test for (A) and (B), one-way ANOVA for (C).

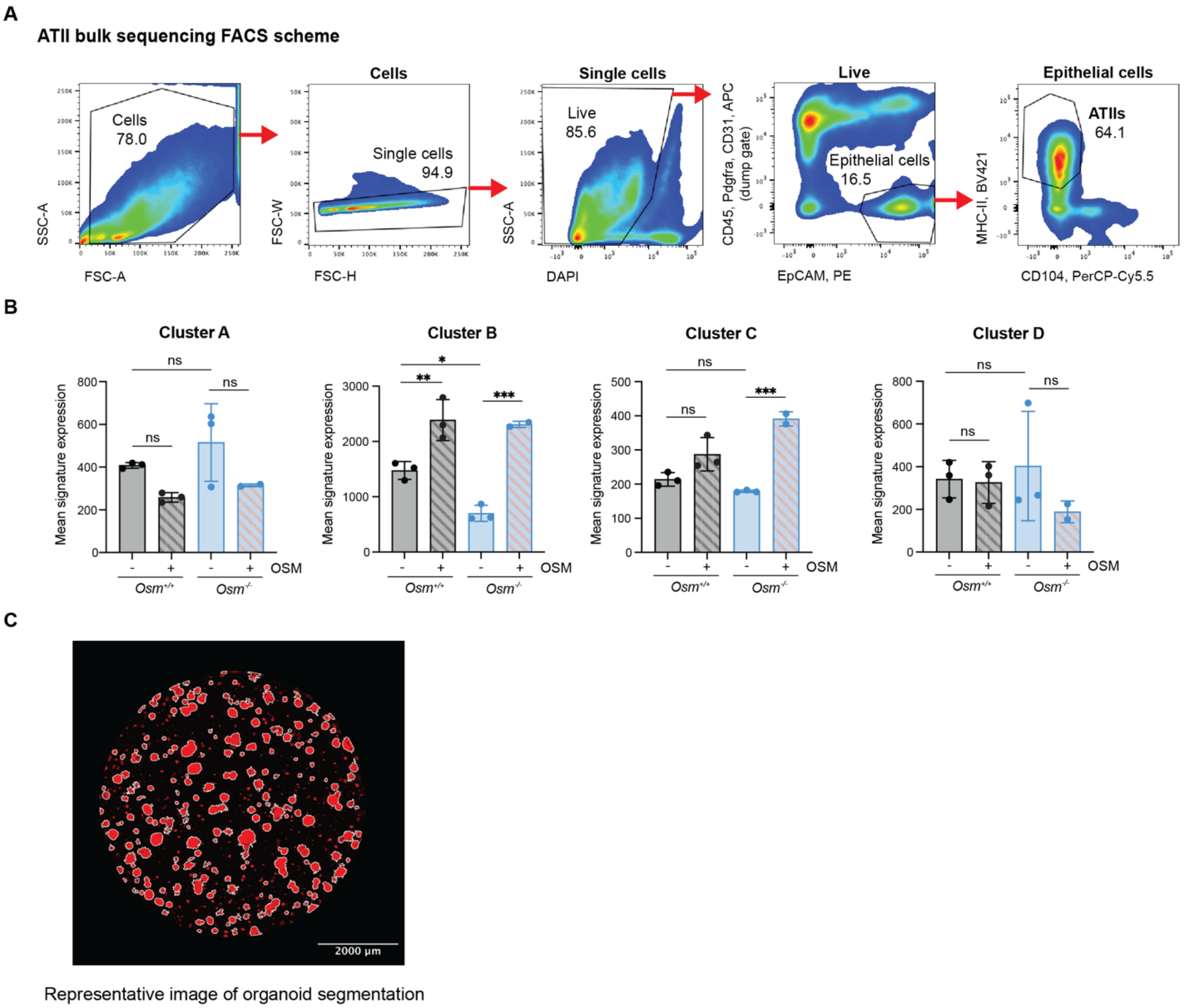

Fig. S17. Pathway enrichment analysis of sorted ATIIs and organoid segmentation. (**A - B**) Mice were i.t. treated with PBS or 1 µg of rOSM for seven consecutive days. Lungs were processed, stained, and ATIIs were sorted as DAPI- CD45- Pdgfra- CD31- EpCAM+ MHCII+ CD104- viable singlets. Representative gating strategy for sorted ATIIs (A). Using *k*-means clusters from Fig. 4D, average expression of genes within each cluster were calculated and compared between groups (B). (**C**) Representative image of alveolar organoid segmentation analysis. Symbols represent individual mice, bars are means. Error bars indicate SD, *p≤0.05, **p≤0.01, ***p≤0.001, ns = not significant. One-way ANOVA for (B).

Supplementary Tables

Table S1: Antibodies

Target marker	Clone	Fluorochrome/Barcode	Concentration	Supplier	RRID	Catalogue number
CD45	30-F11	APC/Cy7	1:200	Biolegend	AB_312980	103115
CD45	30-F11	APC	1:200	Biolegend	AB_312976	103111
CD45	I3/2.3	FITC	1:200	Biolegend	AB_2563541	147709
CD45	30-F11	BV711	1:200	Biolegend	AB_2564383	103147
CD45	30-F11	BUV395	1:200	BD Biosciences	AB_2651134	564279
CD64	X54-5/7.1	APC	1:200	Biolegend	AB_11219391	139306
CD64	X54-5/7.1	PE	1:200	Biolegend	AB_10613467	139303
F4/80	BM8	BV605	1:100	Biolegend	AB_2562305	123133
F4/80	BM8	APC	1:100	Biolegend	AB_893481	123116
F4/80	BM8	BV711	1:100	Biolegend	AB_2564588	123147
F4/80	BM8	Pe/Cy7	1:100	Biolegend	AB_893478	123114
F4/80	BM8	APC/Cy7	1:100	Biolegend	AB_893477	123118
Siglec F	1RNM44N	PE	1:200	eBioscience	AB_2637129	12-1702-82
Siglec F	1RNM44N	Pe/Cy7	1:200	eBioscience	AB_2802251	25-1702-82
Ly6C	HK1.4	BV605	1:500	Biolegend	AB_2562352	128035
Ly6C	HK1.4	PerCP-Cy5.5	1:500	Biolegend	AB_1659242	128011
Ly6C	HK1.4	APC/Cy7	1:500	Biolegend	AB_10643867	128025
Ly6G	1A8	PerCP-Cy5.5	1:300	Biolegend	AB_1877271	127616
Ly6G	1A8	BV785	1:300	Biolegend	AB_2566317	127645

CD11c	N418	APC/Cy7	1:300	Biolegend	AB_830646	117323
CD11c	N418	AF488	1:300	Biolegend	AB_492849	117313
CD11b	M1/70	BV421	1:800	Biolegend	AB_10897942	101235
CD11b	M1/70	PE/Cy7	1:800	Biolegend	AB_312798	101215
I-A/I-E (MHC-II)	M5/114.15.2	BV421	1:400	Biolegend	AB_10900075	107631
I-A/I-E (MHC-II)	M5/114.15.2	AF700	1:400	Biolegend	AB_493727	107622
EPCAM/CD326	G8.8	PE	1:200	Biolegend	AB_1134172	118206
PECAM1/CD31	390	FITC	1:200	Biolegend	AB_312900	102405
PECAM1/CD31	390	APC	1:200	Biolegend	AB_312904	102409
CD104	346-11A	PerCP-Cy5.5	1:200	Biolegend	AB_2734183	123613
PDGFRa/CD140a	APA5	PE/Cy7	1:200	eBioscience	AB_2573400	25-1401-82
PDGFRa/CD140a	APA5	APC	1:200	eBioscience	AB_2784706	47-1401-82
Ki67	B56	BV650	1:100	BD Biosciences	AB_2688008	563757
TCRβ	H57-597	BV605	1:200	BD Biosciences	AB_2687544	562840
CD4	GK1.5	BV421	1:200	Biolegend	AB_10900241	100437
CD8	53 - 6.7	PerCP-Cy5.5	1:200	Biolegend	AB_2075239	100733
FOXP3	FJK-16s	PE	1:100	eBioscience	AB_465936	12-5773-82
B220	RA3-6B2	PE/Cy7	1:200	Biolegend	AB_313004	103221
B220	RA3-6B2	AF488	1:200	Biolegend	AB_389308	103225
NK1.1	PK136	APC/Cy7	1:200	Biolegend	AB_830870	108723
PDCA-1	927	APC	1:200	Biolegend	AB_1967101	127015
pSTAT3	13A3-1	PE	1:100	Biolegend	AB_2571892	651004
Pro-SP-C (SP-C)	EPR19839	N/A	1:1000	Abcam	AB_2927746	ab211326

Uteroglobin (CCSP)	EPR19846	N/A	1:200	Abcam	AB_2650558	ab213203
Donkey anti-rabbit IgG	Poly4064	AF647	1:200	Biolegend	AB_2563202	406414
Hashtag 1 (CD45, MHC-I)	M1/42;30-F11	TotalSeq-B0301	1:50	Biolegend	AB_2814067	155831
Hashtag 2 (CD45, MHC-I)	M1/42;30-F11	TotalSeq-B0302	1:50	Biolegend	AB_2814068	155833

Table S2: Oligonucleotides

Primer	Sequence
mouse *Ifit1* forward	5' - CAACCATGGGAGAGAATGCTG - 3'
mouse *Ifit1* reverse	5' - TGCATCCCCAATGGGTTCTT - 3'
mouse *Irf7* forward	5' - CAGCGAGTGCTGTTTGGAGAC - 3'
mouse *Irf7* reverse	5'- AAGTTCGTACACCTTATGCGG - 3'
mouse *Isg15* forward	5' - AAGCAGCCAGAAGCAGACTC - 3'
mouse *Isg15* reverse	5' - TTAGGTCCCAGGCCATTGCT - 3'
mouse *Ifnb1* forward	5' - GTCTCATTCCACCCAGTGCT - 3'
mouse *Ifnb1* reverse	5' - CAGCTCCAAGAAAGGACGAA - 3'
mouse *Ifna4* forward	5' - CCAGAGAGTGACCAGCATCTAC - 3'
mouse *Ifna4* reverse	5' - AAGGCCCTCTTGTTCCCGAG - 3'
mouse *Osm* forward	5' - GAACACAGAATCACTCTTGG - 3'
mouse *Osm* reverse	5' - TGTCTTAAAGCATCCAGTTG - 3'
mouse *Osmr* forward	5' - CCATAGAGTTCATCCAAAGG - 3'
mouse *Osmr* reverse	5' - GATCACTGAAGAGGTAGTTTG - 3'
mouse *Ifnl2* forward	5' - GAGGTGCAGTTCCCACCTCTTCCC - 3'
mouse *Ifnl2* reverse	5' - CTTCAGGGTCAGGGCCACCTCAG - 3'

NP forward	5′ - GACGATGCAACGGCTGGTCTG - 3′
NP reverse	5′ - ACCATTGTTCCAACTCCTTT - 3′
mouse *Rpl13* forward	5’ - AGTATCTGGCCTTTCTCCGG - 3’
mouse *Rpl13* reverse	5’ - CCGAACAACCTTGAGAGCAG - 3’

Data files S1-S4 are uploaded separately as excel files

Data file S1: Gene list of the top 50 marker genes for each annotated cluster from scRNA-seq. Female mice were infected i.n. with 225 pfu of A/WSN/1933 (H1N1) or mock-infected with PBS. Lungs were collected from IAV-infected mice at 2 dpi and mock-infected mice (0 dpi) for scRNA-seq analysis (n = 2 mice per group). Markers are ranked by adjusted p-value across all clusters.

Data file S2: Gene list and Reactome pathway analysis for ATII subclusters. Female mice were infected i.n. with 225 pfu of A/WSN/1933 (H1N1) or mock-infected with PBS. Lungs were collected from IAV-infected mice at 2 dpi and mock-infected mice (0 dpi) for scRNA-seq analysis (n = 2 mice per group). Gene list used for ATII annotation is included, obtained from (*30*). The ATII cluster was further subclustered into: ATII cluster 1 (OSM-independent), ATII cluster 2 (OSM-dependent), and ATII cluster 3 (ISGhi). Genes for each subcluster are filtered by adjusted p-value (<1E-5) and organized in descending order of log2 fold change (log2FC). Reactome pathway enrichment outputs from Enrichr for each set of subcluster defining genes are included as separate tables.

Data file S3: Filtered gene list from whole lung bulk RNA-seq. Female mice were infected i.n. with 225 pfu of A/WSN/1933(H1N1) or mock-infected with PBS. Lungs were collected from IAV-infected mice at 3 and 7 dpi, and from mock-infected mice (0 dpi) for bulk RNA-seq analysis (n = 2 mice per group). Genes were pre-filtered based on a maximum coefficient of variation of 0.7 and a minimum expression threshold of 10.

Data file S4: Sorted ATII bulk RNA-seq gene lists and pathway analysis. Female mice were treated i.t. with PBS or 1 µg of rOSM for seven consecutive days. Lungs were processed 24 hours after final treatment, stained, and ATIIs were sorted (representative gating strategy for sorted ATIIs found in fig. S17A) (n = 2 – 3 mice per group). Gene list for sorted ATIIs, pre-filtered using a maximum coefficient of variation of 0.2 and a minimum expression threshold of 10. Genes that exhibited at least a twofold differential expression in any pairwise comparison between groups were clustered using *k*-means based on expression values. MSigDB pathway enrichment outputs from Enrichr for each cluster are included as separate tables.
